# Lifestyle Advice Combined with Personalized Estimates of Genetic or Phenotypic Risk of Type 2 Diabetes, and Objectively Measured Physical Activity: A Randomized Controlled Trial

**DOI:** 10.1371/journal.pmed.1002185

**Published:** 2016-11-29

**Authors:** Job G. Godino, Esther M. F. van Sluijs, Theresa M. Marteau, Stephen Sutton, Stephen J. Sharp, Simon J. Griffin

**Affiliations:** 1 MRC Epidemiology Unit, University of Cambridge School of Clinical Medicine, Cambridge, United Kingdom; 2 Center for Wireless and Population Health Systems, Department of Family Medicine and Public Health and Calit2’s Qualcomm Institute, University of California, San Diego, La Jolla, California, United States of America; 3 Behaviour and Health Research Unit, University of Cambridge School of Clinical Medicine, Institute of Public Health, Cambridge, United Kingdom; 4 Behavioural Science Group, University of Cambridge School of Clinical Medicine, Institute of Public Health, University of Cambridge, Cambridge, United Kingdom; 5 Primary Care Unit, University of Cambridge School of Clinical Medicine, Institute of Public Health, University of Cambridge, Cambridge, United Kingdom; University of Oxford, UNITED KINGDOM

## Abstract

**Background:**

Information about genetic and phenotypic risk of type 2 diabetes is now widely available and is being incorporated into disease prevention programs. Whether such information motivates behavior change or has adverse effects is uncertain. We examined the effect of communicating an estimate of genetic or phenotypic risk of type 2 diabetes in a parallel group, open, randomized controlled trial.

**Methods and Findings:**

We recruited 569 healthy middle-aged adults from the Fenland Study, an ongoing population-based, observational study in the east of England (Cambridgeshire, UK). We used a computer-generated random list to assign participants in blocks of six to receive either standard lifestyle advice alone (control group, *n* = 190) or in combination with a genetic (*n* = 189) or a phenotypic (*n* = 190) risk estimate for type 2 diabetes (intervention groups). After 8 wk, we measured the primary outcome, objectively measured physical activity (kJ/kg/day), and also measured several secondary outcomes (including self-reported diet, self-reported weight, worry, anxiety, and perceived risk). The study was powered to detect a between-group difference of 4.1 kJ/kg/d at follow-up. 557 (98%) participants completed the trial. There were no significant intervention effects on physical activity (difference in adjusted mean change from baseline: genetic risk group versus control group 0.85 kJ/kg/d (95% CI −2.07 to 3.77, *p* = 0.57); phenotypic risk group versus control group 1.32 (95% CI −1.61 to 4.25, *p* = 0.38); and genetic risk group versus phenotypic risk group −0.47 (95% CI −3.40 to 2.46, *p* = 0.75). No significant differences in self-reported diet, self-reported weight, worry, and anxiety were observed between trial groups. Estimates of perceived risk were significantly more accurate among those who received risk information than among those who did not. Key limitations include the recruitment of a sample that may not be representative of the UK population, use of self-reported secondary outcome measures, and a short follow-up period.

**Conclusions:**

In this study, we did not observe short-term changes in behavior associated with the communication of an estimate of genetic or phenotypic risk of type 2 diabetes. We also did not observe changes in worry or anxiety in the study population. Additional research is needed to investigate the conditions under which risk information might enhance preventive strategies. (Current Controlled Trials ISRCTN09650496; Date applied: April 4, 2011; Date assigned: June 10, 2011).

**Trial Registration:**

The trial is registered with Current Controlled Trials, ISRCTN09650496.

## Abstract 2

In a randomized controlled trial, Job Godino and colleagues study the effects of providing personalized information about genetic and phenotypic risk of type 2 diabetes as compared with standard lifestyle advice.

## Author Summary

**Why Was This Study Done?:**

- Despite questions regarding their clinical validity and utility, genetic tests aimed at predicting risk of type 2 diabetes are now widely available.
- Some researchers and direct-to-consumer genetic testing companies are optimistic that personalized genetic information will encourage people at risk to adopt healthier behavior than standard advice about lifestyle, but scientific evidence is needed.

**What Did the Researchers Do and Find?:**

- The researchers calculated the risk of developing type 2 diabetes for 569 healthy middle-aged adults.
- The risk estimates were either based on a participant’s genetic makeup, as judged by presence of genetic information known to be associated with type 2 diabetes, or on phenotypic characteristics, such as sex, age, body mass index, etc.
- Participants were randomly assigned to three groups to receive standard lifestyle advice, alone or in combination with their genetic risk estimate or phenotypic risk estimate. After 8 wk, participants’ physical activity, self-reported diet and weight, anxiety, worry, and beliefs about their risk were measured.
- The researchers found that receipt of a genetic or phenotypic risk estimate did not affect participants’ physical activity or other relevant behaviors, in comparison with those receiving standard lifestyle advice. However, perception of risk became more accurate.

**What Do These Findings Mean?:**

- In this study, provision of personalized information about genetic or phenotypic risk of type 2 diabetes did not affect behavior when compared with standard lifestyle advice.
- Provision of personalized information about risk of type 2 diabetes did not seem to cause anxiety.
- More research is needed to understand the circumstances in which genetic risk information might motivate behavior change.

## Introduction

The prevalence of type 2 diabetes is increasing worldwide, and primary prevention of the disease is a global priority [1]. Evidence from randomized controlled trials shows that positive changes in health behavior can significantly reduce the incidence of type 2 diabetes among those considered high-risk [2,3]. However, translating these findings into preventive strategies has proven difficult, as it requires motivation of individuals to adopt and maintain changes in physical activity and diet [4].

Risk of type 2 diabetes is also influenced by genetics. Despite questions regarding the clinical validity and utility of recently developed predictive genetic tests [5,6], some researchers and direct-to-consumer genetic testing companies are optimistic that the provision of genetic risk information for type 2 diabetes will motivate behavior change more than widely available phenotypic risk information [7–9]. This hypothesis has support from health behavior theory [10,11]. However, there is concern about the potential negative psychological impact of widely available genetic risk information including fatalism, anxiety, and false reassurance [12–14]. Furthermore, there is evidence that risk perceptions and communication of biomarker information have limited influence on behavior [15,16].

There are few clinical studies concerning the impact of genetic risk information [17]. The majority have been nonrandomized or underpowered. Furthermore, interpretation has been limited by risk of bias and additional differences between study groups than merely the provision of DNA-based disease risk information. Two recent trials report no behavioral impact of information about the genetic risk of type 2 diabetes [18,19]. However, neither included precise measures of behavior. In addition, Grant et al. recruited a small sample of individuals willing to participate in an intensive diabetes prevention program [19], and Voils et al. did not include a control group receiving no phenotypic risk information [18].

We examined the effect of communicating genetic or phenotypic risk of type 2 diabetes in combination with standard lifestyle advice on objectively-measured physical activity, self-reported diet, self-reported weight, anxiety, and several cognitive and emotional theory-based antecedents of behavior change in a sample of healthy middle-aged adults.

## Methods

Details regarding the trial methods have been reported previously [20]. We obtained ethical approval from the Cambridgeshire 1 Research Ethics Committee (No. 10/H0304/78). Each participant provided written informed consent. The trial is registered with Current Controlled Trials (ISRCTN09650496; Date applied: April 4, 2011; Date assigned: June 10, 2011).

### Participant Screening and Recruitment

We recruited participants from the Fenland Study, an ongoing population-based observational study investigating the influence of lifestyle and genetic factors on the development of diabetes, obesity, and related metabolic disorders [21]. Individuals born between 1950 and 1975 registered with participating general practices in Cambridgeshire, UK were invited to take part. General practitioners excluded those with a diagnosis of diabetes, a terminal illness with a prognosis of less than one year, a psychotic illness, being pregnant or lactating, or being unable to walk unaided. Fenland Study participants undergo a health assessment, and blood samples are collected for the genotyping of single nucleotide polymorphisms (SNPs) associated with type 2 diabetes. At the end of the assessment, participants are fitted with a combined heart rate monitor and accelerometer (Actiheart) [22], which they are instructed to wear continuously for six days and nights to measure physical activity.

We sent invitations to take part in the Diabetes Risk Communication Trial (DRCT) to Fenland Study participants who 1) had agreed to be contacted regarding future studies, 2) had sufficient data to calculate their genetic and phenotypic risk of type 2 diabetes, 3) wore the combined heart rate monitor and accelerometer for three or more full days without experiencing a severe skin reaction, and 4) provided at least 36 h of complete physical activity data. Upon response, we excluded those who reported being diagnosed with diabetes or actively participating in another study.

### Randomization and Blinding

We randomly allocated eligible participants who completed a baseline questionnaire to one of three groups that received either standard lifestyle advice alone (control group) or in combination with a genetic or a phenotypic risk estimate for type 2 diabetes (intervention groups). A statistician without knowledge of participant characteristics created a computer-generated list comprised of blocks of six that contained two of each of the three study groups per block in a random order. This was incorporated into an automated randomization computer program. Allocation was concealed from the researchers and participants until the interventions were assigned. Researchers assessing the primary outcome remained blinded to the allocation of participants throughout the study.

### Interventions

All participants received standard written lifestyle advice, which included a brief description of type 2 diabetes and an explanation of the risk factors, symptoms, diagnosis, treatment, and consequences of the disease. We informed participants that the disease is preventable and encouraged them to maintain a healthy weight and to adhere to United Kingdom governmental guidelines for physical activity and diet [23,24].

The interventions were designed to incorporate evidence regarding the most effective methods for communicating disease risk estimates [25]. As it remains unclear whether an individual’s understanding of risk is more accurate after the provision of a numerical risk estimate or a verbal risk estimate [26,27], both the genetic and phenotypic risk estimates included estimates of the participant’s lifetime risk of developing type 2 diabetes expressed as a percentage and verbal estimation of risk (i.e., “below average,” “average,” or “above average”). Moreover, research suggests that comparative risk estimates may have a greater influence on behavior than absolute risk estimates [28,29], and that visual representations of risk elicit greater recall and understanding of risk [26,30,31]. Thus, estimates were framed in comparison to the average risk within each participant’s age and sex-specific group, and participants were told what percentage of the study sample had a risk estimate higher, lower, and equal to their own. Each piece of information was represented using a visual scales [27].

Methods for calculating the genetic and phenotypic risk estimates have been described in detail previously [20]. We calculated genetic risk using methods similar to those outlined by several direct-to-consumer genetic testing companies [32–34]. The 23 SNPs utilized in the calculation were identified through adequately powered genome-wide association studies, had associations with type 2 diabetes that reached the genome-wide significance level (*p*-values for associations less than 5x10^−8^), and had associations that were replicated in at least one independently published study. We took the odds ratio for each SNP from replication samples and the allele frequency from the HapMap population. We calculated phenotypic risk using the Cambridge Diabetes Risk Score [35,36]. Age, sex, smoking status, family history of diabetes, and prescription of steroid or antihypertensive medication were assessed via questionnaire. We measured height and weight using standardized procedures and calculated body mass index as weight (in kg) divided by the square of height (in m). All data used in the estimation of risk were collected during the participant’s Fenland Study health assessment.

### Outcome Measures

The primary outcome was physical activity, defined as physical activity energy expenditure (kJ/kg/d), measured objectively using the Actiheart continuously for six days and night [22]. We used a submaximal exercise test for individual calibration of heart rate response [37] and a branched equation model to estimate physical activity energy expenditure from acceleration and heart rate [38]. This approach has high validity for estimating the intensity of physical activity [39,40] and overcomes some of the key limitations associated with either accelerometers or heart rate monitors alone [22].

Baseline physical activity was measured in the Fenland Study (median 1.76 years prior to enrollment in the DRCT), and follow-up occurred 8 wk postintervention. This relatively short follow-up period was chosen on the basis that it is unlikely that a long-term effect would exist in the absence of an impact in the short term. All prespecified secondary outcomes were measured via questionnaire and included self-reported diet, self-reported weight, self-rated health, worry (measured at baseline and follow-up), anxiety, behavioral intention, perceived risk, self-efficacy, response efficacy, perceived severity, and diabetes risk representations (measured at baseline, immediately post receipt of the intervention, and follow-up; more details are available in S1 Appendix).

### Statistical Analysis

All analyses were performed on an intention-to-treat basis (i.e., analysis of data according to randomized study group, regardless of whether or not the intervention was received) using STATA software [41]. We used univariate descriptive statistics (means, standard deviations [SDs], numbers, and percentages) to summarise the characteristics of the study sample at baseline. We used analysis of covariance to assess differences between groups in physical activity at follow-up, adjusted for baseline. Prespecified exploratory analyses were conducted to examine whether sex, age, body mass index, time since the Fenland Study, and baseline measurements of the trial outcomes moderated the intervention effects on physical activity. A further subgroup analysis explored whether a high or low risk estimate moderated the effect of the type of risk estimate (i.e., genetic or phenotypic) on physical activity. The study protocol and statistical analysis plan specified that the analyses should include only participants with complete postintervention or follow-up data (i.e., a complete case analysis). We included participants with missing baseline data in the analyses using the missing-indicator method [42]. Similar regression procedures were used to examine differences in all secondary outcomes. The acceptability of the interventions was assessed by summarizing recruitment rates, loss to follow-up, and reasons for loss to follow-up. Additionally, differences in responses to questions regarding the perceived accuracy of the risk estimates, as well as the retention and discussion of the risk estimates were examined.

Estimates used in the sample size calculation were taken from the Feedback, Awareness and Behavior (FAB) study, which had a similar sample population and the same primary outcome as proposed here (i.e., physical activity energy expenditure) [43]. The mean (SD) physical activity energy expenditure at follow-up in the FAB study was 46.2 (15.4) kJ/kg/d, and the correlation between baseline and follow-up was high (0.69). After making a Bonferroni adjustment for multiple comparisons in a three-group trial, we determined that in order to detect a between-group difference of 4.1 kJ/kg/d at follow-up (which equates to approximately 20 to 25 min of walking per day), with 98.3% significance and 80% power, approximately 465 participants would need to complete the trial [44,45]. We conservatively allowed for an attrition rate of 20% and targeted the recruitment of 580 participants.

## Results

### Participant Characteristics

**Fig 1:**
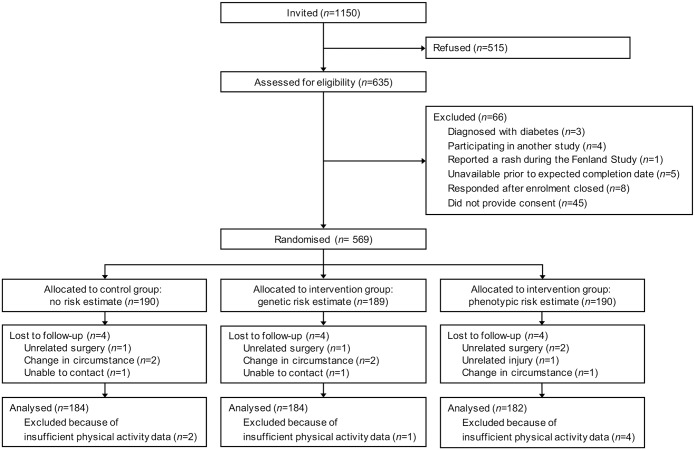
Flow of participants through the DRCT. Baseline characteristics were similar among the three study groups (Table 1). There were slightly more female (52.9%) than male participants. The mean (SD) age at which participants finished full-time education was 19.4 (4.4) y and most were employed full-time (68.0%). Overall, 10.6% were current smokers, and 26.8% consumed more than 11 units of alcohol per wk. Few participants were prescribed steroid or antihypertensive medication (5.8%) or had a positive family history for diabetes (23.0%). On average, participants were overweight (mean [SD] body mass index of 26.1 [4.2] kg/m^2^), but their HbA_1c_ level was in the normal range (mean [SD] of 36.3 [4.4] mmol/mol).

Between February 11, 2011 and September 5, 2011, we sent invitations to take part in the DRCT to 1150 Fenland Study volunteers and 635 (55%) replied positively and were assessed for eligibility. Between March 8, 2011 and September 14, 2011, 569 (49%) participants were randomized (Fig 1). Reasons for exclusion included not responding after an initial positive reply (68%), responding after enrollment closed (12%), being unavailable prior to the expected trial completion date (8%), reporting a rash while wearing the Actiheart during the Fenland Study (1%), participating in another study (6%), and being diagnosed with diabetes (5%). Those who did not reply and those who were excluded from participation did not differ from those randomized according to sex, body mass index, glycated haemoglobin (HbA_1c_) or phenotypic risk. However, they were slightly younger than participants at the time of their Fenland Study health assessment (mean [SD] age 45.0 [6.9] y versus 47.2 [7.4] y) and had a higher genetic lifetime risk estimate (mean [SD] of 18.8% [8.2%] versus 17.8% [8.1%]).

**Table 1:** Baseline characteristics of participants.

Measure	Control (*n* = 190)	Genetic Risk (*n* = 189)	Phenotypic Risk (*n* = 190)	Total (*n* = 569)
Age—y	48.5±7.0	48.7±7.5	49.0±7.4	48.7±7.3
Male sex—no. (%)	98 (51.6)	79 (41.8)	91 (47.9)	268 (47.1)
Age finished full time education—y	19.2±4.1	19.4±4.1	19.7±5.0	19.4±4.4
Employed full-time—no. (%)	130 (68.8)	121 (65.1)	129 (70.1)	380 (68.0)
Current smoker—no. (%)	21 (11.2)	20 (10.7)	18 (10.0)	59 (10.6)
Consumed more than 11 units of alcohol per wk—no. (%)	51 (26.8)	44 (23.4)	43 (22.6)	138 (24.3)
Prescribed steroid or antihypertensive medication—no. (%)	9 (4.7)	13 (6.88)	11 (5.8)	33 (5.8)
Positive family history of diabetes—no. (%)	46 (24.2)	42 (22.2)	43 (22.6)	131 (23.0)
Weight—kg	76.9±14.4	76.3±14.8	75.1±14.3	76.1±14.5
Height—cm	171.3±9.6	169.9±9.9	170.7±9.4	170.6±9.6
Body mass index—kg/m^2^	26.1±4.2	26.4±4.4	25.7±4.2	26.1±4.2
Waist—cm	89.3±11.9	88.8±12.1	87.5±12.4	88.6±12.2
HbA_1c_ —mmol/mol	36.1±3.8	36.4±3.9	36.4±5.3	36.3±4.4
Fasting glucose—mmol/l	4.7±0.5	4.8±0.5	4.7±0.5	4.7±0.5
Triglycerides—mmol/liter	1.1±0.7	1.1±0.8	1.1±0.7	1.1±0.7
Cholesterol—mmol/liter				
Total	5.3±1.1	5.3±1.1	5.2±1.0	5.3±1.1
Low-density lipoprotein	3.3±0.9	3.3±0.9	3.3±0.9	3.3±0.9
High-density lipoprotein	1.6±0.4	1.6±0.4	1.5±0.3	1.5±0.4
Blood pressure—mm Hg				
Systolic	119.6±13.7	121.4±15.4	120.5±15.3	120.5±14.8
Diastolic	73.4±8.8	74.5±10.2	73.8±10.5	73.9±9.9
Genetic risk—%	18.1±8.7	18.1±8.5	17.3±7.0	17.8±8.1
Phenotypic risk—%	24.1±8.9	24.4±9.2	22.9±9.6	23.8±9.3
Time since the Fenland Study—y	2.0±0.7	1.9±0.6	1.9±0.6	1.9±0.6

*Plus–minus values are means ±SD.

After randomization, 12 (2.1%) participants were lost to follow-up, and we excluded 7 (1.2%) from the primary analysis because they had insufficient physical activity data (the monitor did not record more than three days of data) (Fig 1). The complete case sample comprised 550 participants: 184 received standard lifestyle advice alone, 184 received standard lifestyle advice and a genetic risk estimate, and 182 received standard lifestyle advice and a phenotypic risk estimate.

### Outcomes

**Fig 2:**
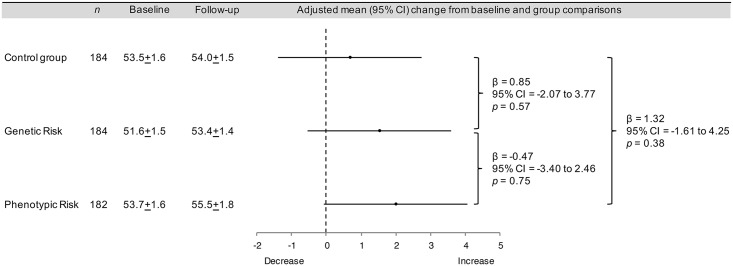
Intervention effects on the primary outcome: physical activity. Physical activity was defined as physical activity energy expenditure (kJ/kg/d) measured with a combined heart rate monitor and accelerometer. Plus–minus values are means ± standard error (SE). Analysis of covariance was used to assess differences between groups at follow-up, adjusted for baseline.

There were no significant between-group differences in objectively measured physical activity at the 8-wk follow-up (Fig 2). Prespecified exploratory analyses showed that the interventions had no effect on physical activity within subgroups defined by age, body mass index, physical activity, self-reported diet, self-reported weight, self-rated health, behavioral intention, perceived risk, anxiety, worry, time since participation in the Fenland Study, or receipt of a high or low risk estimate (Table A in S1 Appendix). However, when compared to the control group, the genetic risk estimate was associated with a greater increase in physical activity among women than among men (women: β = 4.29, 95% CI = 0.27 to 8.33, *p* = 0.037; men: β = −2.69, 95% CI = −6.92 to 1.55, *p* = 0.213). The phenotypic risk estimate did not have a differential effect by sex (women: β = 1.70, 95% CI = −2.45 to 5.86, *p* = 0.421; men: β = 1.33, 95% CI = −2.75 to 5.41, *p* = 0.523).

No significant differences were observed between trial groups in self-reported diet, self-reported weight, self-rated health, and worry at follow-up, nor were there any significant differences in behavioral intention and anxiety immediately postintervention or at follow-up (Tables 2 and 3). Participants who received a risk estimate had a lower perceived risk immediately postintervention than those who did not receive an estimate. These effects were attenuated at follow-up, but remained statistically significant, and did not differ by the type of risk estimate received (Tables 2 and 3). Additionally, immediately postintervention, both diet response efficacy and illness understanding were significantly lower in the phenotypic risk group compared to the control group, and perceived severity was significantly lower in the genetic risk group compared to the control group (Tables 2 and 3).

**Table 2:** Baseline and follow-up values for secondary outcomes by study group.

Measure	Control Group				Genetic Risk Estimate				Phenotypic Risk Estimate			
	*N*	Baseline	Postintervention	Follow-up	*N*	Baseline	Postintervention	Follow-up	*N*	Baseline	Postintervention	Follow-up
Self-reported diet (g/day)	186	383.5±15.1	-	382.1±16.8	183	445.5±16.5	-	403.0±15.4	185	417.5±16.3	-	411.9±17.7
Self-reported weight (kg)	176	77.0±1.09	-	76.7±1.10	180	76.1±1.15	-	75.9±1.13	181	73.9±1.03	-	73.5±1.02
Self-rated health	186	1.99±0.05	-	1.97±0.05	185	1.96±0.05	-	1.95±0.04	184	1.92±0.05	-	1.91±0.04
Worry	185	7.52±0.14	-	7.79±0.15	185	7.54±0.14	-	8.04±0.16	185	7.48±0.14	-	7.84±0.15
Anxiety	162	32.4±0.75	32.7±0.87	31.7±0.79	163	33.2±0.85	32.9±0.88	32.1±0.79	163	33.3±0.91	33.4±0.86	31.8±0.79
Behavioral intention												
Physical activity	182	3.61±0.05	3.72±0.05	3.55±0.05	184	3.56±0.05	3.67±0.05	3.55±0.05	180	3.62±0.05	3.67±0.05	3.55±0.05
Diet	181	3.60±0.05	3.74±0.05	3.61±0.05	183	3.67±0.05	3.75±0.05	3.65±0.05	180	3.54±0.05	3.75±0.05	3.61±0.04
Perceived risk	181	38.7±1.70	41.0±1.76	41.5±1.71	181	39.2±1.81	32.9±1.70	37.7±1.78	181	38.8±1.82	33.4±1.68	36.5±1.74
Self-efficacy												
Physical activity	185	3.93±0.05	3.91±0.05	-	184	3.88±0.05	3.85±0.05	-	182	3.88±0.05	3.84±0.05	-
Diet	185	3.86±0.05	3.87±0.05	-	184	3.96±0.05	3.99±0.05	-	182	3.87±0.05	3.90±0.04	-
Response efficacy												
Physical activity	185	3.88±0.04	4.21±0.05	-	184	3.96±0.04	4.21±0.04	-	182	3.87±0.05	4.11±0.05	-
Diet	185	3.92±0.04	4.22±0.05	-	184	4.04±0.04	4.25±0.04	-	182	3.96±0.05	4.12±0.05	-
Perceived severity	185	3.58±0.06	3.85±0.06	-	184	3.69±0.06	3.78±0.06	-	182	3.52±0.06	3.71±0.06	-
Diabetes risk representations												
Consequences	184	5.41±0.15	6.25±0.15	-	185	5.82±0.16	6.25±0.16	-	182	5.53±0.16	6.07±0.15	-
Timeline	184	8.76±0.15	9.22±0.12	-	185	8.96±0.14	9.15±0.13	-	182	8.69±0.16	9.14±0.12	-
Personal control	185	6.72±0.14	6.78±0.13	-	184	6.45±0.14	6.38±0.15	-	181	6.78±0.15	6.83±0.12	-
Treatment control	185	7.64±0.14	7.67±0.15	-	184	7.49±0.14	7.46±0.15	-	182	7.78±0.13	7.70±0.12	-
Identity	184	5.80±0.13	5.90±0.12	-	184	5.87±0.14	5.99±0.14	-	181	5.90±0.12	5.77±0.13	-
Concern	185	7.06±0.15	7.48±0.14	-	184	7.37±0.17	7.51±0.15	-	182	7.34±0.15	7.41±0.15	-
Understanding	185	4.90±0.18	6.87±0.15	-	184	5.14±0.18	6.79±0.14	-	182	5.26±0.17	6.59±0.15	-
Emotional response	185	5.34±0.16	5.70±0.15	-	184	5.61±0.17	5.78±0.16	-	182	5.43±0.17	5.82±0.16	-

Plus–minus values are means ± SE. Diet was defined as self-reported fruit and vegetable consumption measured via the Food Frequency Questionnaire. Self-rated health was measured on a scale (from 1 to 4), with higher values indicating a poorer rating of health. Worry was measured using an adapted version of the Cancer Worry Scale (from 6 to 24), with higher values indicating greater type 2 diabetes related worry. Anxiety was measured using the short-form of the state scale of the Spielberger State Trait Anxiety Inventory (from 20 to 80), with higher values indicating greater anxiety. Intention was measured on a scale (from 1 to 5), with higher values indicating greater intention to change behavior. Perceived risk was measured on a scale (from 0 to 100), with higher values indicating a greater perceived risk of developing type 2 diabetes. Self-efficacy, response efficacy, and perceived severity were each measured on a scale (from 1 to 5), with higher values indicating a greater likelihood of behavior change. Diabetes risk representations were measured using an adapted Brief Illness Perceptions Questionnaire (from 0 to 10), with higher values indicating that the representation is more important in the participant’s construction of perceived risk.

**Table 3:** Intervention effects on secondary outcomes.

Measure	Differences (95% CI) in adjusted mean change from baseline between groups and corresponding p-values		
	Genetic Risk Estimate versus Control Group	Phenotypic Risk Estimate versus Control Group	Genetic Risk Estimate versus Phenotypic Risk Estimate
Self-reported diet (g/d)	−16.2 (−54.5to 22.0), *p* = 0.40	9.52 (−28.4 to 47.5), *p* = 0.62	−25.8 (−63.9 to 12.4), *p* = 0.19
Self-reported weight (kg)	0.01 (−0.63 to 0.65), *p* = 0.98	−0.31 (−0.95 to 0.33), *p* = 0.35	0.32 (−0.32 to 0.96), *p* = 0.33
Self-rated health	−0.001 (−0.09 to 0.09), *p* = 0.99	−0.02 (−0.11 to 0.07), *p* = 0.71	0.02 (−0.07 to 0.11), *p* = 0.72
Worry	0.24 (−0.09 to 0.56), *p* = 0.16	0.08 (−0.24 to 0.41), *p* = 0.61	0.15 (−0.17 to 0.48), *p* = 0.36
Anxiety			
Postintervention	−0.36 (−2.22 to 1.50), *p* = 0.70	0.10 (−1.74 to 1.95), *p* = 0.91	−0.47 (−2.31 to 1.37), *p* = 0.62
Follow-up	−0.15 (−1.90 to 1.59), *p* = 0.86	−0.40 (−2.14 to 1.34), *p* = 0.65	0.28 (−1.49 to 1.98), *p* = 0.78
Behavioral intention			
Physical activity			
Postintervention	−0.04 (−0.16 to 0.07), *p* = 0.48	−0.05 (−0.17 to 0.06), *p* = 0.38	0.01 (−0.11 to 0.13), *p* = 0.90
Follow-up	0.02 (−0.10 to 0.14), *p* = 0.75	−0.02 (−0.14 to 0.10), *p* = 0.72	0.04 (−0.08 to 0.16), *p* = 0.50
Diet			
Postintervention	−0.03 (−0.14 to 0.08), *p* = 0.57	−0.05 (−0.05 to 0.16), *p* = 0.33	−0.08 (−0.19 to 0.02), *p* = 0.12
Follow-up	−0.01 (−0.12 to 0.09), *p* = 0.80	−0.04 (−0.06 to 0.15), *p* = 0.41	−0.06 (−0.16 to 0.05), *p* = 0.29
Perceived risk			
Postintervention	−8.36 (−12.11 to −4.61), *p* < 0.01	−7.62 (−11.38 to −3.87), *p* < 0.01	−0.74 (−4.49 to 3.01), *p* = 0.70
Follow-up	−4.07 (−7.74 to −0.39), *p* = 0.03	−5.01 (−8.69 to −1.33), *p* < 0.01	0.95 (−2.73 to 4.62), *p* = 0.61
Response efficacy			
Physical activity	−0.04 (−0.15 to 0.07), *p* = 0.44	−0.10 (−0.21 to 0.01), *p* = 0.07	0.06 (−0.05 to 0.17), *p* = 0.30
Diet	−0.03 (−0.14 to 0.07), *p* = 0.52	−0.12 (−0.23 to −0.01), *p* = 0.03	0.09 (−0.21 to 0.19), *p* = 0.12
Self-efficacy			
Physical activity	−0.03 (−0.13 to 0.08), *p* = 0.61	−0.04 (−0.14 to 0.06), *p* = 0.45	0.01 (−0.09 to 0.12), *p* = 0.80
Diet	0.05 (−0.04 to 0.15), *p* = 0.28	0.02 (−0.08 to 0.11), *p* = 0.70	0.03 (−0.06 to 0.13), *p* = 0.49
Perceived severity	−0.14 (−0.27 to −0.01), *p* = 0.04	−0.10 (−0.24 to 0.03), *p* = 0.12	−0.04 (−0.17 to 0.10), *p* = 0.61
Diabetes risk representations			
Consequences	−0.21 (−0.56 to 0.14), *p* = 0.23	−0.24 (−0.60 to 0.11), *p* = 0.17	0.03 (−0.32 to 0.38), *p* = 0.86
Timeline	−0.16 (−0.44 to 0.12), *p* = 0.26	−0.05 (−0.33 to 0.23), *p* = 0.73	−0.11 (−0.39 to 0.17), *p* = 0.44
Personal control	−0.29 (−0.62 to 0.04), *p* = 0.08	0.03 (−0.29 to 0.36), *p* = 0.84	−0.33 (−0.66 to 0.004), *p* = 0.05
Treatment control	−0.13 (−0.48 to 0.22), *p* = 0.47	−0.03 (−0.38 to 0.32), *p* = 0.88	−0.10 (−0.45 to 0.25), *p* = 0.57
Identity	0.06 (−0.26 to 0.37), *p* = 0.73	−0.18 (−0.50 to 0.15), *p* = 0.28	0.23 (−0.09 to 0.55), *p* = 0.16
Concern	−0.16 (−0.48 to 0.17), *p* = 0.34	−0.23 (−0.55 to 0.09), *p* = 0.17	0.07 (−0.25 to 0.39), *p* = 0.66
Understanding	−0.19 (−0.54 to 0.17), *p* = 0.30	−0.44 (−0.80 to −0.09), *p* = 0.02	0.26 (−0.10 to 0.61), *p* = 0.16
Emotional response	−0.09 (−0.42 to 0.24), *p* = 0.58	0.06 (−0.26 to 0.39), *p* = 0.69	−0.16 (−0.49 to 0.17), *p* = 0.34

Diet was defined as self-reported fruit and vegetable consumption measured via the Food Frequency Questionnaire. Self-rated health was measured on a scale (from 1 to 4), with higher values indicating a poorer rating of health. Worry was measured using an adapted version of the Cancer Worry Scale (from 6 to 24), with higher values indicating greater type 2 diabetes-related worry. Anxiety was measured using the short-form of the state scale of the Spielberger State Trait Anxiety Inventory (from 20 to 80), with higher values indicating greater anxiety. Intention was measured on a scale (from 1 to 5), with higher values indicating greater intention to change behavior. Perceived risk was measured on a scale (from 0 to 100), with higher values indicating a greater perceived risk of developing type 2 diabetes. Self-efficacy, response efficacy, and perceived severity were each measured on a scale (from 1 to 5), with higher values indicating a greater likelihood of behavior change. Diabetes risk representations were measured using an adapted Brief Illness Perceptions Questionnaire (from 0 to 10), with higher values indicating that the representation is more important in the participant’s construction of perceived risk. Analysis of covariance was used to assess differences between groups postintervention and at follow-up, adjusted for baseline.

Among those who received a risk estimate, the majority (93.0%) reported that they believed their risk estimate to be either fairly or very accurate. Most participants (90.5%) stated that they had kept their risk estimate, and many (63.7%) reported discussing it with others (for example family members, friends, or health professionals).

## Discussion

In a sample of healthy, middle-aged men and women who were given information about type 2 diabetes and standard lifestyle advice, there was no effect of communicating a genetic or phenotypic estimate of the risk of developing type 2 diabetes on objectively measured physical activity. We did not observe significant intervention effects on self-reported diet and weight, self-rated health, behavioral intentions, anxiety, or worry. This is an important observation, given the expectations that such communications might facilitate behavior change and the concerns about the potential adverse psychological consequences of predictive genetic testing. We also did not observe significant intervention effects on a range of other cognitive and emotional theory-based antecedents to behavior change. We examined several potential moderators, and only sex was found to interact with the intervention effect on physical activity, raising the possibility that genetic risk information may be more influential among women than among men. More research is needed to explore whether women and men respond differently to genetic risk information.

Risk information was received and understood, and had a sustained effect on participants’ perceptions of their risk. However, the volunteers tended to overestimate their risk at baseline and may therefore have been somewhat reassured by the information that they received, albeit not to the extent that they adopted unhealthy behaviors [46]. Nevertheless, we cannot exclude the possibility that provision of risk estimates that exceed participants’ perceived risk might influence behavior, although previous trials have not reported effects among high-risk subgroups [18,19]. Furthermore, it is possible that information concerning the genetic risk of diseases other than diabetes, such as various cancers or chronic neurodegenerative diseases, might elicit a greater response, although this has not been the finding of published trials [47].

This trial provides much needed robust evidence on the behavioral impact of communicating genetic risk information. A systematic review identified only two clinical trials that assessed physical activity and diet as outcomes [47]. The authors concluded that given the limited number of low quality studies, strong conclusions could not be drawn and larger, higher quality studies were needed. The findings of this study suggest that the provision of a genetic risk information, which reduced perceived risk in the majority of participants, did not motivate healthy changes in behavior over and above phenotypic risk information or standard lifestyle advice alone. Findings are consistent with those of a cohort study of the impact of direct-to-consumer genome-wide testing [48] and recent trials of type 2 diabetes genetic risk information [18,19] and add to existing evidence showing that those who undergo testing seldom experience psychological harm [13,49]. While risk information appears not to motivate changes in health behaviors, there is some evidence that it may influence decisions about use of medication [50,51].

Previous research indicates that the effect of communicating genetic risk information on perceived risk, a central construct in many health behavior theories, is unclear. Most studies report that perceived risk decreases after receipt of risk information, usually towards a more accurate perception of risk [52]. We found that the provision of a genetic risk estimate was associated with lower and more accurate perceived risk both immediately and after eight weeks. However, there were no differences in risk perception between those receiving genetic information and a phenotypic risk estimate that can be calculated using routinely collected clinical data. Small but statistically significant immediate effects of risk information on diet response efficacy, illness understanding, and perceived severity may have arisen through multiple testing.

The strengths of this trial include the recruitment of a relatively large sample, a randomized design with sufficient power to assess clinically important impacts on objectively measured behavior, and a high rate of study completion (97%). We presented risk information in a manner similar to that of several direct-to-consumer genetic testing companies. Potential limitations are that participants were recruited from one location in the UK, were well-educated, physically and psychologically healthy, and exhibited limited socioeconomic and ethnic diversity. Consequently, the results might not generalize to other settings or groups. Other limitations include the use of a baseline measure of physical activity that occurred prior to enrollment in the study, self-report questionnaires to measure all secondary outcomes, and a relatively short time to follow-up. However, it is widely assumed that the continuous measure of physical activity for three or more days accurately captures habitual physical activity levels. To the extent that this is true and baseline differences were equally distributed across study groups, the time between the baseline measure of physical activity and enrolment should not have influenced the results. Furthermore, it is unlikely that communication of risk information would have a long-term effect in the absence of an impact in the short term. We did not attempt to assess the clinical validity and utility of the genetic or phenotypic risk estimates as the objective of the trial was to determine the effect of the reported estimates on behavior, regardless of the accuracy of the estimate. However, the risk estimates have been validated in other studies. Although nearly all participants who received a risk estimate believed it to be accurate, the extent to which they were aware of the uncertain clinical validity and utility of predictive genetic tests may have influenced the results of the trial.

In conclusion, we found that communicating an estimate of the risk of type 2 diabetes, either based on genotype or phenotype, did not motivate changes in behavior in the short term, but neither did it cause an increase in worry or anxiety. These findings are consistent with systematic review evidence and should inform the ongoing debate regarding the regulatory response to the proliferation of direct-to-consumer genetic testing companies. Additional research is needed to investigate the conditions under which risk information might enhance preventive strategies. Approaches targeting individual behavior change, such as communicating genetic risk, are unlikely to be successful in isolation in an environment in which there are many impediments to being physically active and eating a healthy diet. The results of the current study thus provide further evidence for a shift in focus for promoting healthy changes in habitual, environmentally patterned behaviors, such as physical activity and diet, away from interventions solely based on provision of information and advice to individuals towards interventions that target the wider collective determinants of disease [53].

## Supporting Information

S1 CONSORT Checklist (DOCX)

S1 Protocol (PDF)

S1 Appendix (DOCX)

## Abbreviations

DRCT: Diabetes Risk Communication Trial
FAB: Feedback, Awareness, and Behavior
HbA_1c_: glycated haemoglobin
SD: standard deviation
SE: standard error
SNP: single nucleotide polymorphism
