# Supplementary material for: Lifestyle Advice Combined with Personalized Estimates of Genetic or Phenotypic Risk of Type 2 Diabetes, and Objectively Measured Physical Activity: A Randomized Controlled Trial
Source: PLoS Med. 2016 Nov 29;13(11):e1002185. doi: 10.1371/journal.pmed.1002185 (PMC5127499; doi:10.1371/journal.pmed.1002185)
Supplement: S1 Protocol — (PDF) [file pmed.1002185.s002.pdf]

BMC Public Health

STUDY PROTOCOL Open Access

Effect of communicating genetic and phenotypic risk for type 2 diabetes in combination with lifestyle advice on objectively measured physical activity: protocol of a randomised controlled trial

Job G Godino[1], Esther MF van Sluijs[1], Theresa M Marteau[2], Stephen Sutton[3], Stephen J Sharp[1] and Simon J Griffin[1*]

Abstract

Background: Type 2 diabetes (T2D) is associated with increased risk of morbidity and premature mortality. Among those at high risk, incidence can be halved through healthy changes in behaviour. Information about genetic and phenotypic risk of T2D is now widely available. Whether such information motivates behaviour change is unknown. We aim to assess the effects of communicating genetic and phenotypic risk of T2D on risk-reducing health behaviours, anxiety, and other cognitive and emotional theory-based antecedents of behaviour change.

Methods: In a parallel group, open randomised controlled trial, approximately 580 adults born between 1950 and 1975 will be recruited from the on-going population-based, observational Fenland Study (Cambridgeshire, UK). Eligible participants will have undergone clinical, anthropometric, and psychosocial measurements, been genotyped for 23 single-nucleotide polymorphisms associated with T2D, and worn a combined heart rate monitor and accelerometer (Actiheart®) continuously for six days and nights to assess physical activity. Participants are randomised to receive either standard lifestyle advice alone (control group), or in combination with a genetic or a phenotypic risk estimate for T2D (intervention groups). The primary outcome is objectively measured physical activity. Secondary outcomes include self-reported diet, self-reported weight, intention to be physically active and to engage in a healthy diet, anxiety, diabetes-related worry, self-rated health, and other cognitive and emotional outcomes. Follow-up occurs eight weeks post-intervention. Values at follow-up, adjusted for baseline, will be compared between randomised groups.

Discussion: This study will provide much needed evidence on the effects of providing information about the genetic and phenotypic risk of T2D. Importantly, it will be among the first to examine the impact of genetic risk information using a randomised controlled trial design, a population-based sample, and an objectively measured behavioural outcome. Results of this trial, along with recent evidence syntheses of similar studies, should inform policy concerning the availability and use of genetic risk information.

Trial registration: Current Controlled Trials ISRCTN09650496

Keywords: Genetic, Phenotypic, Risk, Communication, Type 2 diabetes, Physical activity, Behaviour, Randomised controlled trial, Protocol

* Correspondence: simon.griffin@mrc-epid.cam.ac.uk
[1]MRC Epidemiology Unit, Institute of Metabolic Science, Addenbrooke's Hospital, Hills Road, Box 285, CB2 0QQ, Cambridge, UK
Full list of author information is available at the end of the article

BioMed Central

© 2012 Godino et al.; licensee BioMed Central Ltd. This is an Open Access article distributed under the terms of the Creative Commons Attribution License (http://creativecommons.org/licenses/by/2.0), which permits unrestricted use, distribution, and reproduction in any medium, provided the original work is properly cited.

Background

Type 2 diabetes (T2D) is associated with costly complications including cardiovascular disease, neuropathy, and blindness, as well as premature mortality [1]. Previous research has demonstrated that the development of T2D can be prevented through healthy changes in behaviour, even among those at high risk [2]. More specifically, several randomised controlled trials including individuals with impaired glucose tolerance, have reported reductions in risk by as much as 40% to 60% following interventions to promote moderate weight loss through a combination of changes in physical activity and diet [3].

There are many well-established risk factors for T2D, including demographic (age and sex), metabolic (obesity), and behavioural factors (physical inactivity and poor diet) [1]. Evidence from twin and family history studies has shown that genetic factors also play an important role in determining individual susceptibility [4]. While it has been estimated that between 30% and 70% of T2D risk can be attributed to genetic factors, until recently, the number of genes involved and the extent to which each contributes to the development of the disease remained largely unknown [4,5]. However, with the implementation of large-scale studies of genetic association, our understanding has greatly increased [4,5]. To date, more than 40 single nucleotide polymorphisms (SNPs) associated with increased risk of T2D have been identified [6–12].

Along with the discovery that several genetic loci are associated with T2D came an expectation that this information would lead to improved prediction of disease risk [13–16]. Multiple risk models that incorporate routinely collected data (e.g., sex, age, body mass index, parental history of T2D, drug treatment, and smoking status) have already been shown to predict the risk of developing T2D reasonably well [17,18]. However, contrary to expectations, there currently appears to be little additional predictive benefit from incorporating genetic information into non-genetic risk models [17–19]. A recent meta-analysis of published data on T2D risk prediction showed that the predictive value of SNPs associated with T2D was significantly poorer than the predictive value of non-genetic risk models, with the area under the receiver operating characteristic curve ranging from 0.59 to 0.60 and from 0.78 to 0.89, respectively [19]. Furthermore, when genetic information was incorporated into non-genetic risk models, no clinically significant improvements in the models' predictive values were observed. In spite of these findings, direct-to-consumer genetic tests that claim to predict susceptibility for T2D are now widely available [20], and some researchers are optimistic that this information might enhance preventive strategies [13,21,22].

Consistent with health behaviour theory, it has been suggested that informing individuals of their genetic risk of T2D may motivate engagement in risk-reducing health behaviours (i.e., increased physical activity and a healthy diet) [23,24]. It is thought that genetic risk information could be perceived as more personally relevant than risk information based on other markers of disease. Individuals who are informed that they are at increased risk based on their genotype may consequently have greater motivation to change their behaviour than those informed of increased risk based upon another less salient method (e.g., phenotypic characteristics), or those informed of a low risk. In contrast, it has also been suggested that informing individuals of their genetic risk of T2D might de-motivate some to change their behaviour [24,25]. They could perceive their risk as being uncontrollable due to its genetic determinants, which are often thought to be immutable [26]. Those informed of a high risk as determined by their genotype might consequently adopt a sense of fatalism, and those informed of a low risk might be falsely reassured. In both instances, such attitudes would likely result in a lack of motivation to change behaviour.

In order to take full advantage of the motivational impact of genetic risk information, a deeper understanding of the mechanisms through which it may motivate behaviour change is necessary [23]. Several health behaviour theories, including the Protection Motivation Theory (PMT), indicate that engagement in risk-reducing health behaviours is largely influenced by an individual's pre-existing perception of risk [27,28]. Although there has been much research on the role of perceived risk as a determinant of behaviour, how individuals construct their perception of risk and the extent to which it directly influences behaviour remains unclear [28–30]. The most common method used for assessing perceived risk is through self-report of absolute risk, which is often numerically based [31]. Studies have shown, however, that individuals experience difficulty understanding risk estimates based on numerical presentations of probability or relative risk [32]. Although this may be due in part to problems of numeracy [33,34], some research suggests that it is because individuals construct their perception of risk in ways that are not entirely rational [30,32,35]. The Common Sense Model of self-regulation of health and illness (CSM) provides a framework for understanding how perceived risk is constructed [36]. Previous research highlights the importance of using the CSM as the conceptual basis for identifying the content and influences of perceived risk [32], and also supports examining the utility of the PMT to predict changes in behaviour [37,38]. Together, the CSM and the PMT could elucidate which components of T2D risk information effectively motivate change in risk-reducing health behaviours.

To date, no research has examined the effect of communicating genetic risk of T2D on the risk-reducing health behaviours of physical activity and diet. The

majority of genetic risk communication research has focused on diseases that have single-allele associations and do not have complex gene-lifestyle interactions [39,40]. A recent systematic review by Marteau et al. [39], identified seven randomised controlled trials and six analogue studies that evaluated the effects of providing genetic risk information for a range of diseases (e.g., lung cancer, heart disease, and Alzheimer's disease). The studies explored a variety of behavioural outcomes (e.g., smoking cessation, medication adherence, and vitamin use), many of which were assessed through imprecise self-report measures. Only two of the trials included in the review assessed self-reported changes in physical activity and diet, and neither was in the context of T2D. In line with the findings of similar reviews [41–44], the authors concluded that the limited number of low quality studies precluded strong statements regarding the effect of communicating genetic risk information on non-clinical risk-reducing behaviours. There was no evidence that genetic risk information de-motivated individuals. However, it does appear that genetic risk information may not greatly motivate behaviour change, but may have a small effect on intentions to change behaviour [39]. Given the rapid growth in our understanding of the genetic basis of complex disease and the increasingly widespread availability of genetic tests, there is a pressing need to improve our knowledge of the potential for beneficial or harmful effects of informing people of their genetic risk of disease [45,46].

Objectives

The primary objective of the Diabetes Risk Communication Trial (DRCT) is to assess whether communicating a genetic risk estimate for T2D in combination with standard lifestyle advice motivates greater changes in objectively measured physical activity than a phenotypic risk estimate for T2D in combination with standard lifestyle advice or standard lifestyle advice alone.

The secondary objectives are to determine the effects of the interventions on self-reported diet; self-reported weight; intentions to be physically active and to engage in a healthy diet; anxiety; diabetes-related worry; self-rated health; and other cognitive and emotional theory-based antecedents to behaviour change.

Methods

Study design

The DRCT is a parallel group, open randomised controlled trial with allocation of approximately 580 participants to one of three groups. Participants in each group receive standard lifestyle advice, which includes general information about T2D as well as information about how to reduce the risk of developing the disease. In addition to this information, one group receives a genetic risk estimate for T2D, while another group receives a phenotypic risk estimate (intervention groups). The remaining group of participants (control group) does not receive either of the risk estimates until after they have completed follow-up. The design of the trial and flow of participants are shown in Figure 1.

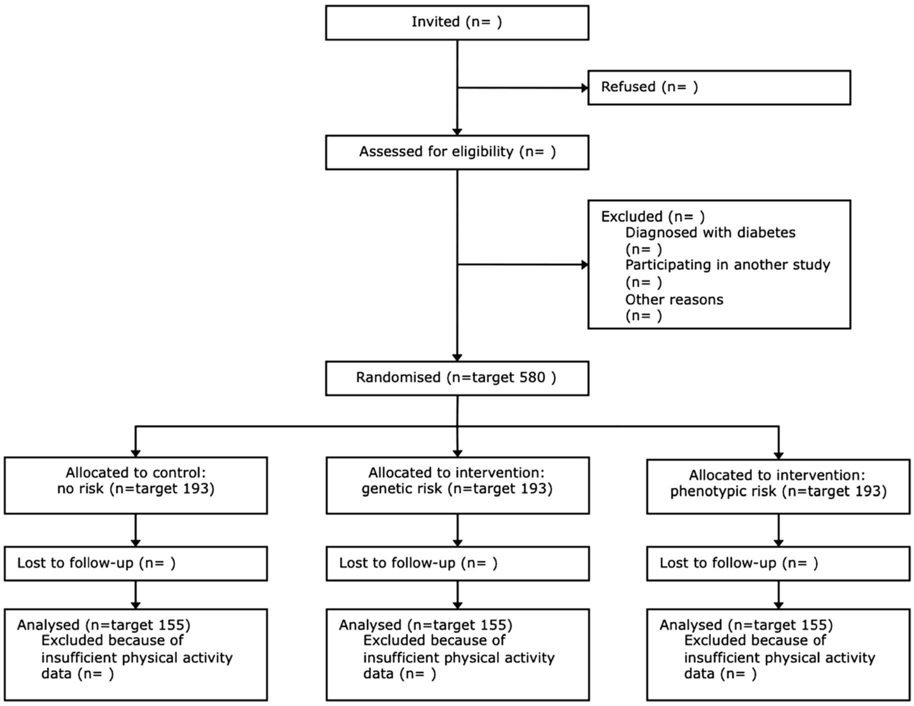

Figure 1 The flow of participants through the Diabetes Risk Communication Trial.

Recruitment

Participants are recruited from the Fenland Study, an ongoing population-based, observational study investigating the influence of lifestyle and genetic factors on the development of diabetes, obesity, and other metabolic disorders [47]. Residents of Cambridgeshire, in the east of England, born between 1950 and 1975 are potentially eligible to participate in the Fenland Study and are excluded by their general practitioner if they have been diagnosed with diabetes or a terminal illness with a prognosis of less than one year. Additionally, they are excluded if they suffer from a psychotic illness, are pregnant or lactating, or if they are unable to walk unaided. Approximately 28% of those registered with participating general practices in the Cambridgeshire Primary Care Trust have enrolled in the Fenland Study (more than 8,000 participants). Fenland Study participants visit a study centre where they undergo a health assessment. Blood samples are taken for the genotyping of SNPs associated with T2D. At the end of the assessment, Fenland Study participants are fitted with a combined heart rate monitor and accelerometer (described in detail below), which they are instructed to wear continuously for six days and nights prior to returning it to the study centre.

Participants of the Fenland Study are invited to take part in the DRCT if they have given permission to be contacted regarding potential involvement in future studies. Furthermore, they must have provided sufficient data to calculate their genetic and phenotypic risk estimates for T2D (described in detail below), and have worn the combined heart rate monitor and accelerometer for three or more full days without experiencing a severe skin reaction. Lastly, the monitor must have recorded at least 36 hours of complete data. Fenland Study participants who fulfil the inclusion criteria are invited to take part in the DRCT in the order in which they completed their health assessment (median 1.76 years prior to being invited), beginning with the most recent first. Potential participants are mailed an invitation letter, a study brochure, a sample consent form, and a response form. Those interested in taking part are asked to complete the response form and to return it in the enclosed freepost reply envelope. In the event of non-response, a second mailing is sent approximately four weeks after the first. Responders are excluded from the DRCT if they have been diagnosed with diabetes or are actively participating in another study. The exclusion criteria are explicitly stated in both the invitation letter and the study brochure, and are assessed in the response form via self-report.

Baseline assessment

All baseline information is collected prior to randomisation. Responders who are eligible to take part in the DRCT are sent a baseline instruction letter, a consent form, and a baseline questionnaire. The instruction letter asks responders to read and sign the consent form prior to completing the baseline questionnaire. They are then asked to return both documents in the enclosed freepost reply envelope. A reminder letter, along with a second consent form and baseline questionnaire, is sent if the first consent form and questionnaire are not returned within two weeks. The measures assessed in the baseline questionnaire are combined with several measures taken during the participants' Fenland Study health assessment in order to create a comprehensive baseline characterisation of the study population.

Randomisation, allocation concealment and blinding

In order to ensure equal allocation across the three trial groups, participants are allocated to one of the groups using a blocked randomisation procedure. A statistician without knowledge of participant characteristics created a computer-generated list comprised of blocks of six that contain two of each of the three trial groups per block in a random order. The randomisation list was incorporated into a computer program that members of the study coordination team use for enrolment and automated randomisation of participants. Allocation is concealed from the study coordination team, researchers, and participants until the interventions are assigned. It is not possible to blind participants to which intervention they receive. However, researchers assessing the baseline characteristics of participants and the primary outcome of the trial remain blinded to group assignment. Additionally, an independent, quality assured data-entry company undertakes all data entry unaware of group allocation.

Intervention

The interventions consist of either a genetic risk estimate or a phenotypic risk estimate for T2D, both in combination with standard lifestyle advice (Additional file 1: Appendices A through C for examples). Several modes of intervention delivery were considered, including face-to-face counselling, telephone conversations, and printed materials. Printed materials were chosen as they have the advantage of being simple, inexpensive, and do not create any undue burden for participants in relation to time and travel. Both interventions contain information presented in a manner similar to that of several direct-to-consumer genetic testing companies (e.g., www.navigenics.com, www.decodeme.com, and www.23andme.com), and they were designed to incorporate evidence regarding the most effective methods for communicating disease risk estimates. As it remains unclear whether an individual's understanding of risk is more accurate after the provision of a numerical risk estimate or a verbal risk estimate [34,48], both the genetic and phenotypic risk estimates include a numerical (i.e., percentage) and verbal estimation of risk (i.e., "below

average", "average", or "above average"). Moreover, research suggests that comparative risk estimates may have a greater influence on behaviour than absolute risk estimates [49,50], and that visual representations of risk elicit greater recall and understanding of risk [33,34,51]. Thus, estimates are framed in comparison to the average risk within each participant's age and sex specific group, and participants are told what percentage of Fenland Study participants have a risk estimate higher, lower, and equal to their own. Each piece of information is represented using a visual image.

Genetic risk estimate

Trained personnel at a study centre took blood samples during the Fenland Study. Genomic DNA was extracted from up to 7.5 ml of whole blood stabilised in EDTA using an Autopure LS DNA preparation platform (Qiagen, Crawley, UK). Genotyping was carried out by the Department of Pathology, University of Cambridge using the HumanCardio-Metabo Beadchip Kit supplied by Illumina® (Cambridge, UK). The following 23 SNPs were identified through adequately powered genome-wide association studies, the associations with T2D reached the genome-wide significance level (p-values for associations less than $5x10^{-8}$), and the associations were replicated in at least one independently published study: CDKN2A/B *rs10811661*, MTNR1B *rs10830963*, HHEX *rs1111875*, ZFAND6 *rs11634397*, ADCY5 *rs11708067*, SLC30A8 *rs13266634*, CENDT2 *rs1552224*, DGKB/TMEM195 *rs2191349*, KCNQ1 *rs231362*, ADAM30 *rs2641348*, PROX1 *rs340874*, IGF2BP2 *rs4402960*, ADAMTS9 *rs4607103*, ZBED3 *rs4457053*, CDKN2A/B *rs564398*, NOTCH2 *rs10923931*, IRS1 *rs7578326*, CDKAL1 *rs7756992*, GCKR *rs780094*, TCF7L2 *rs7903146*, JAZF1 *rs864745*, TP53INP1 *rs896854*, and VEGFA *rs9472138* [6–12]. All SNPs were in Hardy-Weinberg equilibrium ($\chi 2$, $P > 0.05$). Occasionally, one or more may have been missing due to random error in the genotyping process. In these cases, linkage disequilibrium was taken advantage of in order to impute the genotype at the loci for which data were missing. The odds ratio for each SNP included in the estimation of genetic risk was taken from replication samples, and the allele frequency was taken from the HapMap population.

The genetic risk estimate is presented as an estimate of the participant's lifetime risk of developing T2D. It was calculated using the aforementioned genetic data and procedures outlined in literature published by several direct-to-consumer genetic testing companies (Additional file 1: Appendix D for an example) [52–54]. First, assuming a multiplicative model, the odds ratio for the risk allele of each SNP associated with T2D and the corresponding population frequency of the risk allele were used to determine the odds ratios and frequencies of the three possible genotypes at each locus. Next, the average population risk at each locus, relative to the no risk genotype, was calculated by summing the product of the genotype specific odds ratios and frequencies. Each genotype specific odds ratio was then divided by the average population risk at each locus in order to derive an estimate of the genotype specific risk that is relative to the average population risk at each locus. The genotype specific risks were then combined using a multiplicative model to create a total genetic risk relative to the population for each participant. Lastly, the participant's total genetic risk was multiplied by their corresponding age and sex specific T2D residual lifetime risk estimate [55].

Phenotypic risk estimate

Age, sex, family history of diabetes, smoking status, and prescription of steroid or anti-hypertensive medication were assessed through self-report during the Fenland Study. Height and weight were also measured using standardised procedures [47], and body mass index was calculated as weight (kg) divided by the square of height (m) [56].

The phenotypic risk estimate is presented as an estimate of the participant's lifetime risk of developing T2D. It was calculated using the aforementioned phenotypic data and the Cambridge Diabetes Risk Score [57], which has been previously validated in the EPIC-Norfolk study, where it was shown to provide good prediction of incident T2D (area under the receiver operating characteristic curve equal to 0.75) [58]. First, each participant's T2D risk score was calculated (from 0 to 1) using the published beta-coefficients from the Cambridge Diabetes Risk Score. Next, the natural logarithm of the score was taken and the results were stratified into age and sex specific groups. Lastly, the percentage difference from the mean was calculated for each participant and the resulting percentage was multiplied by their corresponding age and sex specific T2D residual lifetime risk estimate [55]. Calculating the phenotypic risk estimates in this way generated values that were broadly similar to the genetic risk estimates.

Standard lifestyle advice

All participants receive general information about T2D irrespective of risk and group assignment. The information includes a brief description of T2D and an explanation of the risk factors, symptoms, diagnosis, treatment, and consequences of the disease. All participants are told that the likelihood of developing the disease can be reduced by following physical activity and dietary guidelines. They are equally encouraged to maintain a healthy weight and to follow government recommendations to engage in at least 30 minutes of moderate intensity physical activity on five or more days of the week [59] and to eat five servings of fruit and vegetables a day [60].

Immediate post-intervention assessment

Following allocation to a trial group, participants are mailed an intervention instruction letter, standard lifestyle advice

along with the relevant intervention materials, and an immediate post-intervention questionnaire. The instruction letter asks participants to read through the intervention materials until they feel happy that they understand them, complete the immediate post-intervention questionnaire, and return it in the enclosed freepost reply envelope. A reminder letter, along with a second copy of the questionnaire, is sent if the first is not returned within two weeks. If the questionnaire is still not returned after four weeks, a second reminder letter is sent.

Follow-up assessment

Approximately eight weeks post-intervention, participants are sent a follow-up instruction letter, a combined heart rate monitor and accelerometer, and follow-up questionnaires. A member of the study team contacts each participant by telephone approximately one week before the monitor is due to be posted to check that it is a convenient time for them to wear it. If it is an inconvenient time, an alternative time is arranged. The instruction letter asks participants to wear the monitor for six days and nights continuously, and then return it along with the follow-up questionnaires in the enclosed Special Delivery freepost reply envelope. A reminder letter is sent if responses are not received within two weeks. If responses are still not received after three weeks, a member of the study team contacts the participant by telephone to arrange for the monitor to be returned. If necessary, a second telephone call is made. Upon receipt of the monitor and questionnaires, participants are mailed a study completion letter and a wait-list response form. The response form asks participants to indicate if they would like to be sent whichever risk estimate(s) they have not yet received, and indicates that we will send feedback about their current physical activity level shortly. They are asked to return the response form in the enclosed freepost reply envelope. If a response is not received within two weeks, we send only the physical activity feedback.

Measures

The primary outcome is objectively measured physical activity, defined as physical activity energy expenditure (PAEE, measured in kJ/kg/day). It is assessed using the Actiheart®, a single-piece monitor capable of measuring acceleration, heart rate, heart rate variability, and ECG amplitude for a set time resolution [61]. A sub-maximal exercise test was conducted as part of the Fenland Study and is used for individual calibration of heart rate response [62]. Branched equation modelling is utilised to estimate PAEE [63]. This approach has high validity for estimating the intensity of physical activity [64,65] and overcomes some of the key limitations associated with either accelerometers or heart rate monitors alone [61]. Participants are instructed to wear the monitor for six days and nights continuously, and to carry on with all normal activities during this time. The device is non-invasive, weighs less than 8 g and is worn on the chest attached to standard ECG electrodes stuck directly onto the skin. It is only 7 mm thick (33 mm in diameter), and except for a brief period to change electrodes (every few days) it does not need to be removed. Monitors are also waterproof and can be worn while swimming or showering. These factors make it convenient and discreet to wear. Participants are also asked to complete an Actiheart® log sheet, which indicates the date and time they a) started wearing the monitor, b) removed it (along with the reason) and replaced it again, and c) completed measurement.

All secondary outcomes are measured via self-report questionnaire and are described in detail in Table 1. They include self-reported diet, self-reported weight, intentions to be physically active and to engage in a healthy diet, anxiety, diabetes-related worry, self-rated health, and other cognitive and emotional outcomes that are based on the PMT and the CSM.

Statistical analysis

We will use univariate descriptive statistics (means, standard deviations, numbers, and percentages) to summarise the characteristics of the study sample at baseline. All trial analyses will be performed on an intention-to-treat basis (i.e., analysis of data according to randomised study group, regardless of whether or not the intervention was received). Those with missing outcome data will be excluded from the analyses (a complete case approach). If necessary, a sensitivity analysis will be performed to investigate the effect of having excluded participants with missing data for the primary outcome only. We will use a multiple imputation procedure with a 'missing at random' assumption to impute missing outcome data [89]. Participants with missing baseline data will be included in the analyses using the missing-indicator method [90].

An analysis of covariance (ANCOVA) regression model will be used to determine if differences exist in mean PAEE at follow-up, adjusted for baseline, between randomised groups. Exploratory analyses will be conducted to examine potential moderators and mediators of the intervention effects on PAEE (i.e., sex, age, body mass index, time since the Fenland Study, and baseline measurements of the trial outcomes). A further subgroup analysis will explore whether a high or low risk estimate moderates the effect of type of T2D risk estimate (i.e., genetic or phenotypic) on PAEE. Similar regression analyses will be used to examine differences in all secondary outcomes. Acceptability will be assessed by summarising recruitment rates, loss to follow-up, and reasons for loss to follow-up. Additionally, differences in

Table 1 Measures used in the Diabetes Risk Communication Trial (DRCT)

Measure(s)	Brief description	Stage assessed			
		Fenland study	DRCT baseline	DRCT post-intervention	DRCT follow-up
Demographic characteristics	Sex, age, race/ethnicity, immigrant status, level of education, employment status, and level of income were assessed through self-report.	✓			
Anthropometric, body composition, clinical, physical activity, biochemical, medical history, and lifestyle	Anthropometric (e.g., height, weight, hip and waist), body composition (e.g., precise body fat percentage and distribution using ultrasound and DEXA), clinical (e.g., blood pressure and pulse rate), and physical activity measurements (e.g., heart rate, movement, and oxygen consumption at rest and during a sub-maximal treadmill test) were assessed by trained staff. An oral glucose tolerance test was administered, and two blood samples were taken to assess glucose levels and blood lipids. Medical history and general lifestyle were assessed through self-report.	✓			
Perceived healthy weight	Participants are asked what they think a healthy weight is for them in either stones or kilograms. This measure has been used in previous research [66].		✓		
Perceived weight status	Participants are asked if they think that they are underweight, overweight, or an acceptable weight. This measure has been used in previous research [67,68]		✓		
Perception of diet	1) Participants are asked how much fruit and vegetables they think that they eat compared to people of their age and sex, and answer on a 5-point response scale, ranging from “much less” to “much more”. 2) Participants are asked whether or not they meet the national recommendations for fruit and vegetable consumption. Similar measures have been used in previous research [69,70].		✓		
Perception of physical activity	1) Participants are asked how physically active they think that they are compared to people of their age and sex, and answer on a 5-point response scale, ranging from “much less” to “much more”. 2) Participants are asked whether or not they meet the national guidelines for engagement in physical activity. Similar measures have been used in previous research [71,72].		✓		
History of genetic testing	Participants are asked if they have ever had a genetic test to assess their risk of developing a disease, and if so, to list the disease(s) for which their risk was assessed.		✓		
Process measures	Participants are asked what they think that their risk estimate showed, how accurate they think that their risk estimate is, whether or not they have kept their risk estimate, and whether or not they have discussed their risk estimate with someone. Additionally, participants are asked if they previously had a genetic test to assess their risk of developing a disease, and if so, to list the disease(s) for which their risk was assessed.				✓
Diabetes risk representations*	Assessed using the Brief Illness Perceptions Questionnaire (Brief IPQ) [73]. The Brief IPQ consists of 8 items that address the cognitive and emotional illness representations in the CSM. To capture representations of T2D risk held by healthy individuals, the items have been adapted according to methods used in previous research [74,75]. The Brief IPQ has been shown to have good test-retest reliability and to be highly correlated with relevant subscales of the IPQ-R [73].		✓	✓	
Self-efficacy, response efficacy, and perceived severity*	Assessed using 10 Likert items. Each item includes a statement (e.g., “I am confident that I could be more physically active if I wanted to”) evaluated on a 5-point response scale, ranging from “strongly disagree” to “strongly agree”. These items have been adapted for use in the context of T2D [76,77] and have been used in previous research [78].		✓	✓	
Perceived risk*	1) Participants are asked how likely they think that they are to get T2D in the next 10 years and their lifetime, and first		✓	✓	✓

Table 1 Measures used in the Diabetes Risk Communication Trial (DRCT) *(Continued)*

	answer on a 5-point response scale, ranging from “very unlikely” to “very likely”, and then on a continuous scale, ranging from 1 to 100. 2) Participants are asked how likely they think they are to get T2D in the next 10 years and their lifetime, compared to people their same age and sex, and answer on a 5-point response scale, ranging from “much less likely” to “much more likely”. These items have been adapted according to recommendations provided by Diefenbach et al. [79], and have been used in previous research [80].				
Self-rated health*	Participants are asked if they think that their overall health is excellent, good, fair, or poor. This measure has been used in previous research [81].		✓		✓
Diabetes-related worry*	Assessed using the Cancer Worry Scale (CWS) [82]. The CWS consists of 6 items that assess the frequency of worries about developing cancer and the effect that these worries have on mood and daily functioning. These items have been adapted for use in the context of T2D and have been shown to have acceptable test-retest reliability and good internal consistency [83].		✓		✓
Anxiety*	Assessed using the short-form of the state scale of the Spielberger State Trait Anxiety Inventory (STAI) [84]. The short-form STAI consists of 6 items that comprise the most highly correlated anxiety-present and anxiety-absent items from the full-form of the STAI. Scores obtained using this short-form have been shown to be highly correlated with scores obtained using the full-form of the STAI [84].		✓	✓	✓
Intentions to be physically active and engage in a healthy diet*	Assessed using 4 items. Each item includes a statement (e.g., “I intend to be more physically active in the next 8 weeks.”) evaluated on a 5-point response scale, ranging from “extremely unlikely” to “extremely likely”. These items have been adapted according to recommendations provided by Ajzen [77] and have been used in previous research [78,85].		✓	✓	✓
Self-reported weight*	Participants are asked what their current weight is, without shoes, in either stones or kilograms. Detailed descriptions of the reliability and validity of self-reported weight have been published elsewhere [86].		✓		✓
Self-reported diet*	Assessed using the Food Frequency Questionnaire (FFQ) [87]. The FFQ contains a list of 130 foods, including 12 fruit items and 26 vegetable items. Only the fruit and vegetable items are assessed at follow-up. Detailed descriptions of the reliability and validity of the FFQ have been published elsewhere [87,88].	✓			✓

*Secondary outcomes.

responses to questions regarding the perceived accuracy of the risk estimates, as well as the retention and discussion of the risk estimates will be examined.

Sample size

Estimates used in the sample size calculation were taken from the FAB study, which had a similar sample population and the same primary outcome to that proposed here (i.e., PAEE) [91]. The mean (standard deviation) PAEE at follow-up in the FAB study was 46.2 (15.4) kJ/kg/day, and the correlation between baseline and follow-up was high (0.69). After making a Bonferroni adjustment for multiple comparisons in a three group trial, we determined that in order to detect a between-group difference of 4.1 kJ/kg/day in PAEE at follow-up (which equates to approximately 20 to 25 minutes of brisk walking per day), with a significance level of 1.67% and 80% statistical power, approximately 465 participants will need to complete the trial. Thus, allowing for an attrition rate of 20%, we aim to randomise approximately 580 participants.

Data management and quality assurance

Each participant is assigned a unique numeric identifier at the beginning of the Fenland Study so that they can be tracked without reference to personal information. A new identifier is assigned to participants enrolled in the DRCT, and a data manager uninvolved in data collection maintains a link to the corresponding Fenland Study identifier. All personal data are stored on an encrypted drive, and links to personal information are only available to the study coordination team. Consent forms and questionnaire data are stored in locked filing cabinets in

secure Entacard – protected sites. Questionnaire data are double entered by an independent, quality assured data-entry company.

Trained personnel conduct the trial according to the standard operating procedures established by the MRC Epidemiology Unit and the principles of good clinical practice [92]. Standardised delivery of the interventions is assured by having one member of the study coordination team prepare the intervention materials using an automated computer program. A second member verifies that the intervention materials are correct prior to sending them to participants.

Ethics

Full ethical approval for the Fenland Study was obtained from the Cambridge Local Research Ethics Committee on the 11th of May 2004 (reference number 04/Q0108/19). Full ethical approval for the DRCT was obtained from the Cambridgeshire 1 Research Ethics Committee on the 21st of October 2010 (reference number 10/H0304/78). Written informed consent is obtained from all participants, and each participant's general practitioner is notified of their enrolment.

Discussion

To enhance our understanding of the effects of communicating information about genetic and phenotypic risk of T2D on risk-reducing health behaviours, larger and higher quality randomised controlled trials are needed [39]. Such trials will have the power to detect small, but still clinically important, effect sizes while allowing for a more comprehensive analysis of potential mediators and moderators of effects of risk communication. Studies should include precise measures of behaviours and behavioural intentions, a comprehensive assessment of individual risk perception, and a sound theoretical framework so as to further elucidate the motivational impact of risk communication. Each of these recommendations is consistent with the MRC Framework for the development and evaluation of complex interventions to improve health [93], and highlight the strengths of this study.

Previous research has demonstrated that healthy changes in behaviour can significantly reduce the incidence of T2D, even among those at high risk [2]. However, translation of these findings into preventive strategies has proven difficult, as it requires that individuals are motivated to adopt and maintain the behavioural changes necessary to prevent T2D [94]. The DRCT will provide robust evidence of the potential for beneficial or harmful effects of communicating genetic and phenotypic risk information on risk-reducing health behaviours and the potential role of such information in T2D preventive strategies.

Additional file

Additional file 1: Appendix A. Results booklet containing standard lifestyle advice. **Appendix B.** Example genetic risk estimate. **Appendix C.** Example phenotypic risk estimate. **Appendix D.** Example calculation of the genetic risk estimate.

Abbreviations

T2D: Type 2 Diabetes; SNP(s): Single Nucleotide Polymorphism(s); PMT: Protection Motivation Theory; CSM: Common Sense Model; DRCT: Diabetes Risk Communication Trial; PAEE: Physical Activity Energy Expenditure.

Competing interests

The authors declare that they have no competing interests.

Authors' contributions

JGG, EMFvS, TMM, SS, and SJG, defined the research question; established the design of the trial; and developed the interventions and measures. JGG created the study materials and is responsible for implementing the protocol. SJS assisted with the randomisation procedure and statistical analysis plan. JGG and SJG drafted the manuscript. All authors have read and approved the final manuscript.

Acknowledgements

This trial is funded by The Medical Research Council (MC_U106179474), and is conducted at the MRC Epidemiology Unit in Cambridge, UK. Protocol development was supported by the European Union (Integrated Project LSHM-CT-2006-037197 in the Framework Programme 6 of the European-Community). SJG receives support from the Department of Health NIHR Programme Grant funding scheme (RP-PG-0606-1259). We are grateful to Dr. Roman Pfister for his help extracting data on the SNPs utilised in the calculation of the genetic risk estimates. We thank all staff from the MRC Epidemiology Functional Group Team in particular for study coordination, data collection, physical activity data processing, anthropometry data processing, data management, associated laboratory work, business operations, IT, and research governance. We also thank the Cambridge University Hospitals NHS Foundation Trust Department of Clinical Biochemistry and the NIHR Cambridge Biomedical Research Centre, Core Biochemical Assay Laboratory for carrying out the biochemical assays.

Author details

[1]MRC Epidemiology Unit, Institute of Metabolic Science, Addenbrooke's Hospital, Hills Road, Box 285, CB2 0QQ, Cambridge, UK. [2]Behaviour and Health Research Unit, Institute of Public Health, University of Cambridge, Forvie Site, Robinson Way, CB2 0SR, Cambridge, UK. [3]Behavioural Science Group, Institute of Public Health, University of Cambridge, Forvie Site, Robinson Way, CB2 0SR, Cambridge, UK.

Received: 29 May 2012 Accepted: 30 May 2012
Published: 18 June 2012

References

1. Petit WA, Adamec CA: *The encyclopedia of diabetes*. New York: Facts On File; 2011.
2. Ahmad LA, Crandall JP: **Type 2 diabetes prevention: a review.** *Clin Diabetes* 2010, **28:**53–59.
3. Qi L, Hu FB, Hu G: **Genes, environment, and interactions in prevention of type 2 diabetes: a focus on physical activity and lifestyle changes.** *Curr Mol Med* 2008, **8:**519–532.
4. Doria A, Patti ME, Kahn CR: **The emerging genetic architecture of type 2 diabetes.** *Cell Metab* 2008, **8:**186–200.
5. Bonnefond A, Froguel P, Vaxillaire M: **The emerging genetics of type 2 diabetes.** *Trends Mol Med* 2010, **16:**407–416.
6. Dupuis J, Langenberg C, Prokopenko I, Saxena R, Soranzo N, Jackson AU, Wheeler E, Glazer NL, Bouatia-Naji N, Gloyn AL, *et al*: **New genetic loci implicated in fasting glucose homeostasis and their impact on type 2 diabetes risk.** *Nat Genet* 2010, **42:**105–116.
7. Lyssenko V, Nagorny CLF, Erdos MR, Wierup N, Jonsson A, Spegel P, Bugliani M, Saxena R, Fex M, Pulizzi N, *et al*: **Common variant in MTNR1B**

associated with increased risk of type 2 diabetes and impaired early insulin secretion. *Nat Genet* 2009, **41:**82–88.
8. Saxena R, Voight BF, Lyssenko V, Burtt NP, de Bakker PIW, Chen H, Roix JJ, Kathiresan S, Hirschhorn JN, Daly MJ, *et al*: **Genome-wide association analysis identifies loci for type 2 diabetes and triglyceride levels.** *Science* 2007, **316:**1331–1336.
9. Steinthorsdottir V, Thorleifsson G, Reynisdottir I, Benediktsson R, Jonsdottir T, Walters GB, Styrkarsdottir U, Gretarsdottir S, Emilsson V, Ghosh S, *et al*: **A variant in CDKAL1 influences insulin response and risk of type 2 diabetes.** *Nat Genet* 2007, **39:**770–775.
10. Voight BF, Scott LJ, Steinthorsdottir V, Morris AP, Dina C, Welch RP, Zeggini E, Huth C, Aulchenko YS, Thorleifsson G, *et al*: **Twelve type 2 diabetes susceptibility loci identified through large-scale association analysis.** *Nat Genet* 2010, **42:**579–589.
11. Zeggini E, Scott LJ, Saxena R, Voight BF, Marchini JL, Hu T, de Bakker PIW, Abecasis GR, Almgren P, Andersen G, *et al*: **Meta-analysis of genome-wide association data and large-scale replication identifies additional susceptibility loci for type 2 diabetes.** *Nat Genet* 2008, **40:**638–645.
12. Zeggini E, Weedon MN, Lindgren CM, Frayling TM, Elliott KS, Lango H, Timpson NJ, Perry JRB, Rayner NW, Freathy RM, *et al*: **Replication of genome-wide association signals in UK samples reveals risk loci for type 2 diabetes.** *Science* 2007, **316:**1336–1341.
13. Collins FS, Green ED, Guttmacher AE, Guyer MS: **A vision for the future of genomics research: A blueprint for the genomic era.** *Nature* 2003, **422:**835–847.
14. Grant RW, Moore AF, Florez JC: **Genetic architecture of type 2 diabetes: Recent progress and clinical implications.** *Diabetes Care* 2009, **32:**1107–1114.
15. Majithia AR, Florez JC: **Clinical translation of genetic predictors for type 2 diabetes.** *Curr Opin Endocrinol Diabetes and Obesity* 2009, **16:**100–106.
16. Stolerman ES, Florez JC: **Genomics of type 2 diabetes mellitus: implications for the clinician.** *Nat Rev Endocrinol* 2009, **5:**429–436.
17. Buijsse B, Simmons RK, Griffin SJ, Schulze MB: **Risk assessment tools for identifying individuals at risk of developing type 2 diabetes.** *Epidemiologic Rev* 2011, **33:**46–62.
18. Noble D, Mathur R, Dent T, Meads C, Greenhalgh T: **Risk models and scores for type 2 diabetes: systematic review.** *Br Med J* 2011, **343:**d7163.
19. Talmud PJ, Hingorani AD, Cooper JA, Marmot MG, Brunner EJ, Kumari M, Kivimäki M, Humphries SE: **Utility of genetic and non-genetic risk factors in prediction of type 2 diabetes: Whitehall II prospective cohort study.** *Br Med J* 2010, **340:**b4838.
20. Edelman E, Eng C: **A practical guide to interpretation and clinical application of personal genomic screening.** *Br Med J* 2009, **339:**b4253.
21. Wang C, Bowen DJ, Kardia SL: **Research and practice opportunities at the intersection of health education, health behavior, and genomics.** *Health Educ Behav* 2005, **32:**686–701.
22. Feero WG, Guttmacher AE, Collins FS: **The Genome Gets Personal—Almost.** *JAMA* 2008, **299:**1351–1352.
23. Marteau TM: **Communicating genetic risk information.** *Br Med Bull* 1999, **55:**414–428.
24. Marteau TM, Weinman J: **Self-regulation and the behavioural response to DNA risk information: A theoretical analysis and framework for future research.** *Social Sci Med* 2006, **62:**1360–1368.
25. Collins RE, Wright AJ, Marteau TM: **Impact of communicating personalized genetic risk information on perceived control over the risk: a systematic review.** *Genet Med* 2011, **13:**273.
26. Bates BR, Templeton A, Achter PJ, Harris TM, Condit CM: **What does "a gene for heart disease" mean? A focus group study of public understandings of genetic risk factors.** *Am J Med Genet A* 2003, **119A:**156–161.
27. Boer H, Seydel E: **Protection motivation theory**. In *Predicting Health Behavior 1st edition*. Edited by Connor M, Norman P. Buckingham: Open University Press; 1996:95–118.
28. Brewer N, Weinstein N, Cuite C, Herrington J: **Risk perceptions and their relation to risk behavior.** *Ann Behav Med* 2004, **27:**125–130.
29. Rothman AJ, Kiviniemi MT: **Treating people with information: an analysis and review of approaches to communicating health risk information.** *J Natl Cancer Inst Monogr* 1999, **1999:**44–51.
30. Weinstein ND: **What does it mean to understand a risk? Evaluating risk comprehension.** *J Natl Cancer Inst Monogr* 1999, **1999:**15–20.
31. Price H: **Risk communication: Why, what and how?A discussion of the evidence regarding risk communication and how best to do it in practice.** *Diabetes and Primary Care* 2010, **12:**100.
32. Sivell S, Elwyn G, Gaff CL, Clarke AJ, Iredale R, Shaw C, Dundon J, Thornton H, Edwards A: **How risk is perceived, constructed and interpreted by clients in clinical genetics, and the effects on decision making: Systematic review.** *J Genet Couns* 2008, **17:**30–63.
33. Edwards A, Elwyn G, Mulley A: **Explaining risks: turning numerical data into meaningful pictures.** *Br Med J* 2002, **324:**827–830.
34. Gigerenzer G, Edwards A: **Simple tools for understanding risks: from innumeracy to insight.** *Br Med J* 2003, **327:**741–744.
35. Cameron LD, Sherman KA, Marteau TM, Brown PM: **Impact of genetic risk information and type of disease on perceived risk, anticipated affect, and expected consequences of genetic tests.** *Heal Psychol* 2009, **28:**307.
36. Leventhal H, Brissette I, Leventhal EA: **The common-sense model of self-regulation of health and illness**. In *The self-regulation of health and illness behaviour*. Edited by Cameron LD, Leventhal H. New York: Routledge; 2003:42–65.
37. Floyd D, Prentice-Dunn S: **A meta-analysis of research on protection motivation theory.** *J Appl Soc Psychol* 2000, **30:**407–429.
38. Milne S, Sheeran P, Orbell S: **Prediction and intervention in health-related behavior: A meta-analytic review of protection motivation theory.** *J Appl Soc Psychol* 2000, **30:**106–143.
39. Marteau TM, French DP, Griffin SJ, Prevost A, Sutton S, Watkinson C, Attwood S, Hollands GJ: **Effects of communicating DNA-based disease risk estimates on risk-reducing behaviours.** *Cochrane Database Syst Rev* 2010.
40. McBride CM, Koehly LM, Sanderson SC, Kaphingst KA: **The behavioral response to personalized genetic information: Will genetic risk profiles motivate individuals and families to choose more healthful behaviors?** *Annu Rev Public Health* 2010, **31:**89–103.
41. Marteau TM, Lerman C: **Genetic risk and behavioural change.** *Br Med J* 2001, **322:**1056–1059.
42. Beery TA, Williams JK: **Risk reduction and health promotion behaviors following genetic testing for adult-onset disorders.** *Genet Test* 2007, **11:**111–123.
43. Heshka JT, Palleschi C, Howley H, Wilson B, Wells PS: **A systematic review of perceived risks, psychological and behavioral impacts of genetic testing.** *Genet Med* 2008, **10:**19–32.
44. Scheuner MT, Sieverding P, Shekelle PG: **Delivery of genomic medicine for common chronic adult diseases: a systematic review.** *JAMA* 2008, **299:**1320–1334.
45. McBride CM, Brody LC: **Point: genetic risk feedback for common disease time to test the waters.** *Cancer Epidemiol Biomarkers Prev* 2007, **16:**1724–1726.
46. Khoury MJ, Gwinn M, Yoon PW, Dowling N, Moore CA, Bradley L: **The continuum of translation research in genomic medicine: how can we accelerate the appropriate integration of human genome discoveries into health care and disease prevention?** *Genet Med* 2007, **9:**665.
47. De Lucia Rolfe E, Loos RJF, Druet C, Stolk RP, Ekelund U, Griffin SJ, Forouhi NG, Wareham NJ, Ong KK: **Association between birth weight and visceral fat in adults.** *Am J Clin Nutr* 2010, **92:**347.
48. Edwards A, Gray J, Clarke A, Dundon J, Elwyn G, Gaff C, Hood K, Iredale R, Sivell S, Shaw C, Thornton H: **Interventions to improve risk communication in clinical genetics: Systematic review.** *Patient Educ Counseling* 2008, **71:**4–25.
49. Fagerlin A, Zikmund-Fisher BJ, Ubel PA: **"If I'm better than average, then I'm ok?": Comparative information influences beliefs about risk and benefits.** *Patient Educ Counseling* 2007, **69:**140–144.
50. Mason D, Prevost AT, Sutton S: **Perceptions of absolute versus relative differences between personal and comparison health risk.** *Heal Psychol* 2008, **27:**87.
51. Cameron LD: **Can our health behaviour models handle imagery-based processes and communications?** *The Eur Health Psychologist* 2009, **11:**56–58.
52. Navigenics: *The science behind the Navigenics service*; [http://www.navigenics.com/static/pdf/Navigenics-TheScience.pdf].
53. deCODEme: *Risk calculations*; [http://demo.decodeme.com/health-watch-information/risk-calculation].
54. 23andMe: *(White Paper 23–01) Estimating genotype-specific incidence for one or several loci*; [https://23andme.https.internapcdn.net/res/pdf/HIC-SXIYiYqXreldAxO5yA_23-01_Estimating_Genotype_Specific_Incidence.pdf].
55. Narayan KMV, Boyle JP, Thompson TJ, Sorensen SW, Williamson DF: **Lifetime Risk for Diabetes Mellitus in the United States.** *JAMA* 2003, **290:**1884–1890.
56. NHLBI Obesity Education Initiative Expert Panel on the Identification Evaluation and Treatment of Obesity in Adults: *Clinical guidelines on the identification, evaluation, and treatment of overweight and obesity in adults – The evidence report*. Washington DC: National Institutes of Health; 1998.

57. Griffin S, Little P, Hales C, Kinmonth A, Wareham N: **Diabetes risk score: towards earlier detection of type 2 diabetes in general practice.** *Diabetes Metab Res Rev* 2000, **16**:164–171.
58. Rahman M, Simmons RK, Harding AH, Wareham NJ, Griffin SJ: **A simple risk score identifies individuals at high risk of developing Type 2 diabetes: a prospective cohort study.** *Fam Pract* 2008, **25**:191–196.
59. DOH: *At least five a week: Evidence on the impact of physical activity and its relationship to health*. London: London Department of Health; 2004.
60. Cardiovascular Review Group Committee on Medical Aspects of Food Policy: *Nutritional aspects of cardiovascular disease: Report on health and social subjects*. London: HMSO; 1994.
61. Brage S, Brage N, Franks P, Ekelund U, Wareham N: **Reliability and validity of the combined heart rate and movement sensor Actiheart.** *Eur J Clin Nutr* 2005, **59**:561–570.
62. Brage S, Ekelund U, Brage N, Hennings MA, Froberg K, Franks PW, Wareham NJ: **Hierarchy of individual calibration levels for heart rate and accelerometry to measure physical activity.** *J Appl Physiol* 2007, **103**:682.
63. Brage S, Brage N, Franks P, Ekelund U, Wong M, Andersen L, Froberg K, Wareham N: **Branched equation modeling of simultaneous accelerometry and heart rate monitoring improves estimate of directly measured physical activity energy expenditure.** *J Appl Physiol* 2004, **96**:343.
64. Thompson D, Batterham A, Bock S, Robson C, Stokes K: **Assessment of low-to-moderate intensity physical activity thermogenesis in young adults using synchronized heart rate and accelerometry with branched-equation modeling.** *J Nutr* 2006, **136**:1037.
65. Strath SJ, Brage S, Ekelund U: **Integration of physiological and accelerometer data to improve physical activity assessment.** *Med Sci Sports Exercise* 2005, **37**:S563–S571.
66. Godino JG, Lepore SJ, Rassnick S: **Relation of misperception of healthy weight to obesity in urban black men.** *Obesity* 2009, **18**:1318–1322.
67. Atlantis E, Barnes EH, Ball K: **Weight status and perception barriers to healthy physical activity and diet behavior.** *Int J Obes* 2008, **32**:343–352.
68. Maximova K, McGrath JJ, Barnett T, O'Loughlin J, Paradis G, Lambert M: **Do you see what I see? Weight status misperception and exposure to obesity among children and adolescents.** *Int J Obes* 2008, **32**:1008–1015.
69. Lechner L, Brug J, De Vries H: **Misconceptions of fruit and vegetable consumption: Differences between objective and subjective estimation of intake.** *J Nutr Educ* 1997, **29**:313–320.
70. Lechner L, Brug J, De Vries H, van Assema P, Mudde A: **Stages of change for fruit, vegetable and fat intake: consequences of misconception.** *Heal Educ Res* 1998, **13**:1-a–11.
71. Lechner L, Bolman C, Van Dijke M: **Factors related to misperception of physical activity in The Netherlands and implications for health promotion programmes.** *Heal Promot Int* 2006, **21**:104–112.
72. van Sluijs E, Griffin S, van Poppel M: **A cross-sectional study of awareness of physical activity: associations with personal, behavioral and psychosocial factors.** *Int J Behav Nutr Phys Act* 2007, **4**:53.
73. Broadbent E, Petrie KJ, Main J, Weinman J: **The brief illness perception questionnaire.** *J Psychosom Res* 2006, **60**:631–637.
74. Figueiras MJ, Alves NC: **Lay perceptions of serious illnesses: An adapted version of the Revised Illness Perception Questionnaire (IPQ-R) for healthy people.** *Psychol Heal* 2007, **22**:143–158.
75. Cameron LD: **Illness risk representations and motivations to engage in protective behavior: The case of skin cancer risk.** *Psychol Heal* 2008, **23**:91–112.
76. Ajzen I: **The theory of planned behavior.** *Organ Behav Hum Decis Process* 1991, **50**:179–211.
77. Ajzen I: **Constructing a TpB questionnaire: Conceptual and methodological considerations.** *aa* aa, **aa**:aa. http://socgeo.ruhosting.nl/html/files/spatbeh/tpb.measurement.pdf.
78. Blue CL: **Does the theory of planned behavior identify diabetes-related cognitions for intention to be physically active and eat a healthy diet?** *Public Health Nursing* 2007, **24**:141–150.
79. Diefenbach MA, Weinstein ND, O'Reilly J: **Scales for assessing perceptions of health hazard susceptibility.** *Heal Educ Res* 1993, **8**:181–192.
80. Lipkus IM, Kuchibhatla M, McBride CM, Bosworth HB, Pollak KI, Siegler IC, Rimer BK: **Relationships among breast cancer perceived absolute risk, comparative risk, and worries.** *Cancer Epidemiol Biomarkers Prev* 2000, **9**:973–975.
81. Fayers PM, Sprangers MAG: **Understanding self-rated health.** *Lancet* 2002, **359**:187–188.
82. Watson M, Lloyd S, Davidson J, Meyer L, Eeles R, Ebbs S, Murday V: **The impact of genetic counselling on risk perception and mental health in women with a family history of breast cancer.** *Br J Cancer* 1999, **79**:868.
83. Rees G, Fry A, Cull A, Sutton S: **Illness perceptions and distress in women at increased risk of breast cancer.** *Psychol Heal* 2004, **19**:749–765.
84. Marteau TM, Bekker H: **The development of a six-item short-form of the state scale of the Spielberger State-Trait Anxiety Inventory (STAI).** *Br J Clin Psychol* 1992, **31**:301–306.
85. Park P, Simmons RK, Prevost AT, Griffin SJ: **Screening for type 2 diabetes is feasible, acceptable, but associated with increased short-term anxiety: a randomised controlled trial in British general practice.** *BMC Publ Health* 2008, **8**:350.
86. Gorber SC, Tremblay M, Moher D, Gorber B: **A comparison of direct vs. self-report measures for assessing height, weight and body mass index: a systematic review.** *Obes Rev* 2007, **8**:307–326.
87. McKeown NM, Day NE, Welch AA, Runswick SA, Luben RN, Mulligan AA, McTaggart A, Bingham SA: **Use of biological markers to validate self-reported dietary intake in a random sample of the European Prospective Investigation into Cancer United Kingdom Norfolk cohort.** *Am J Clin Nutr* 2001, **74**:188–196.
88. Bingham SA, Gill C, Welch A, Day K, Cassidy A, Khaw KT, Sneyd MJ, Key TJA, Roe L, Day NE: **Comparison of dietary assessment methods in nutritional epidemiology: weighed records v. 24 h recalls, food-frequency questionnaires and estimated-diet records.** *Br J Nutr* 1994, **72**:619–643.
89. Sterne JAC, White IR, Carlin JB, Spratt M, Royston P, Kenward MG, Wood AM, Carpenter JR: **Multiple imputation for missing data in epidemiological and clinical research: potential and pitfalls.** *Br Med J* 2009, **338**:b2393.
90. White IR, Thompson SG: **Adjusting for partially missing baseline measurements in randomized trials.** *Stat Med* 2005, **24**:993–1007.
91. Watkinson C, van Sluijs E, Sutton S, Marteau T, Griffin S: **Randomised controlled trial of the effects of physical activity feedback on awareness and behaviour in UK adults: the FAB study protocol [ISRCTN92551397].** *BMC Publ Health* 2010, **10**:144.
92. ICH harmonised tripartite guideline: *Guideline for good clinical practice E6 (R1)*. [http://www.ich.org/fileadmin/Public_Web_Site/ICH_Products/Guidelines/Efficacy/E6_R1/Step4/E6_R1__Guideline.pdf].
93. Craig P, Dieppe P, Macintyre S, Michie S, Nazareth I, Petticrew M: **Developing and evaluating complex interventions: the new Medical Research Council guidance.** *Br Med J* 2008, **337**:a1655.
94. Yates T, Davies M, Khunti K: **Preventing type 2 diabetes: can we make the evidence work?** *Postgrad Med J* 2009, **85**:475–480.

doi:10.1186/1471-2458-12-444
Cite this article as: Godino *et al.*: Effect of communicating genetic and phenotypic risk for type 2 diabetes in combination with lifestyle advice on objectively measured physical activity: protocol of a randomised controlled trial. *BMC Public Health* 2012 12:444.

Submit your next manuscript to BioMed Central and take full advantage of:

- **Convenient online submission**
- **Thorough peer review**
- **No space constraints or color figure charges**
- **Immediate publication on acceptance**
- **Inclusion in PubMed, CAS, Scopus and Google Scholar**
- **Research which is freely available for redistribution**

Submit your manuscript at
www.biomedcentral.com/submit

BioMed Central
