# Supplementary material for: Lifestyle Advice Combined with Personalized Estimates of Genetic or Phenotypic Risk of Type 2 Diabetes, and Objectively Measured Physical Activity: A Randomized Controlled Trial
Source: PLoS Med. 2016 Nov 29;13(11):e1002185. doi: 10.1371/journal.pmed.1002185 (PMC5127499; doi:10.1371/journal.pmed.1002185)
Supplement: S1 Appendix — (DOCX) [file pmed.1002185.s003.docx]

**SUPPLEMENTARY APPENDIX**

Lifestyle advice combined with personalized estimates of genetic or phenotypic risk of type 2 diabetes, and objectively measured physical activity: a randomized controlled trial

Job G Godino^1,2^, Esther MF van Sluijs^1^, Theresa M Marteau^3^, Stephen Sutton^4^, Stephen J Sharp^1^ and Simon J Griffin^1,5^

1. MRC Epidemiology Unit, University of Cambridge School of Clinical Medicine, Box 285 Institute of Metabolic Science, Cambridge Biomedical Campus, Cambridge, CB2 0QQ, United Kingdom
2. Center for Wireless and Population Health Systems, Department of Family Medicine and Public Health and Calit2’s Qualcomm Institute, University of California, San Diego, 9500 Gilman Drive, Dept. 0811, La Jolla, CA 92093
3. Behaviour and Health Research Unit, University of Cambridge School of Clinical Medicine, Institute of Public Health, Forvie Site, Robinson Way, Cambridge, CB2 0SR, United Kingdom
4. Behavioural Science Group, University of Cambridge School of Clinical Medicine, Institute of Public Health, University of Cambridge, Forvie Site, Robinson Way, Cambridge, CB2 0SR, United Kingdom
5. Primary Care Unit, University of Cambridge School of Clinical Medicine, Institute of Public Health, University of Cambridge, Forvie Site, Robinson Way, Cambridge, CB2 0SR, United Kingdom

**Measures**

All baseline data was collected prior to randomization. It consisted of measures taken during the Fenland Study and after agreement to take part in the Diabetes Risk Communication Trial. During the Fenland Study, participants completed detailed questionnaires to assess demographic characteristics, medical history, and general lifestyle. They also completed a range of anthropometric (e.g., height, weight, hip and waist), clinical (e.g., blood pressure), and physical activity measurements (e.g., heart rate, movement, and oxygen consumption at rest and during a sub-maximal treadmill test). An oral glucose tolerance test was also administered, and blood samples were taken to assess glucose levels and blood lipids. This information was used to characterize the population at baseline and to calculate each participant’s genetic and phenotypic risk estimates.

Self-reported diet was the only secondary outcome with a baseline measurement taken during the Fenland Study. The baseline measurement of all the other secondary outcomes described below was assessed via questionnaire, after agreement to take part in the Diabetes Risk Communication Trial.

*Self-reported diet*. Diet was defined as fruit and vegetable consumption and was assessed using the 12 fruit items and 26 vegetable items from the Food Frequency Questionnaire (FFQ). Participants were asked to indicate their usual rate of consumption of each food on a 9-point response scale, ranging from never or <1 time per month to >6 times per day. A medium serving of each item was described in terms of units or common portions (e.g., one apple) or household measures (e.g., one glass). Blank spaces were provided for participants to indicate any foods that they consumed more than once a week, but are not listed. Each participant’s responses to the individual food items was converted to an average daily intake of each in grams per day. Detailed descriptions of the reliability and validity of the FFQ have been published elsewhere.

*Self-reported weight.* Self-reported weight was assessed by asking participants, “What is your current weight without shoes?” Participants were given the option to respond in either kilograms or stones and pounds. All responses in stones and pounds were converted to kilograms during data analysis. Detailed descriptions of the reliability and validity of self-reported weight have been published elsewhere.

*Self-rated health.* Self-rated health was assessed by asking participants, “All in all, would you say your overall health is excellent, good, fair, or poor?” Poorer perception of health on this measure has been shown to be associated with greater risk of atherosclerosis, poorer lung functioning, lower exercise capacity and increased mortality.

*Worry.* Worry was assessed using an adapted version of the Cancer Worry Scale (CWS), originally derived from the Lerman Cancer Worry Scale, and adapted here for use in the context of diabetes. The CWS consists of 6 items that assess the frequency of worries about developing diabetes and the impact that these worries have on mood and daily functioning, e.g., “How often do you worry about developing diabetes?” Participants were asked to respond to each item on a 4-point response scale. Scores were summed to create a total score, ranging from 6-24, with higher scores indicating higher levels of diabetes-related worry. The CWS has been shown to have acceptable test-retest reliability and good internal consistency.

*Anxiety.* Anxiety was assessed using the short-form of the state scale Spielberger State Anxiety Inventory (STAI). This version of the STAI consists of 6 items that comprise the most highly correlated anxiety-present and anxiety-absent items from the full-form of the STAI, e.g., “I am worried.” Participants were asked to evaluate each statement on a 4-point response scale, ranging from “not at all” to “very much”. Scores obtained using this short-form have been shown to be highly correlated (r > .90) with scores obtained using the full-form of the STAI.

*Intention.* Physical activity and diet intentions were each assessed using 2 items. Each item included a statement (e.g., “I intend to be more physically active in the next 8 weeks.”), and participants were asked to evaluate each statement on a 5-point response scale (e.g., “extremely unlikely”, “unlikely”, “neither likely nor unlikely”, “likely”, or “extremely likely”). These items have been used in previous research.

*Perceived Risk*. Perceived risk was assessed by asking participants, “On a scale from 0 to 100, where 0 = certain not to happen, and 100 = certain to happen, how likely are you to get type 2 diabetes in your lifetime?” These item was adapted for use in the context of diabetes and has been used in previous research.

*Response efficacy.* Physical activity and diet response efficacy were each assessed using 2 Likert items. Each item included a statement (e.g., “Being physically active is effective in preventing type 2 diabetes”), and participants were asked to evaluate each statement on a 5-point response scale. Response options include “strongly disagree”, “disagree”, “neither agree nor disagree”, “agree”, and “strongly agree”. These items were adapted for use in the context of diabetes and have been used in previous research.

*Self-efficacy.* Physical activity and diet self-efficacy were each assessed using 2 Likert items. Each item included a statement (e.g., “I am confident that I could be physically active if I wanted to”), and participants were asked to evaluate each statement on a 5-point response scale. Response options include “strongly disagree”, “disagree”, “neither agree nor disagree”, “agree”, and “strongly agree”. These items were adapted for use in the context of diabetes and have been used in previous research.

*Perceived severity.* Perceived severity was assessed using 2 Likert items. Each item included a statement (e.g., “I believe that type 2 diabetes is extremely harmful”), and participants were asked to evaluate each statement on a 5-point response scale. Response options include “strongly disagree”, “disagree”, “neither agree nor disagree”, “agree”, and “strongly agree”. These items were adapted for use in the context of diabetes and have been used in previous research.

*Diabetes Risk Representations.* Diabetes risk representations were assessed using the Brief Illness Perceptions Questionnaire (Brief IPQ). The Brief IPQ consists of 9 items that address the cognitive and emotional illness representations in Leventhal’s self-regulatory model. In order to capture representations of type 2 diabetes risk held by healthy individuals, the first 8 items were adapted according to methods used in previous research. Five of the items assess cognitive representations: consequences (“If you had type 2 diabetes, how much would the illness affect your life?”), timeline (“If you got type 2 diabetes, how long do you think the illness would continue?”), personal control (“If you got type 2 diabetes, how much control do you feel you would have over the illness?”), treatment control (“If you got type 2 diabetes, how much do you think treatment could help the illness?”), and identity (“If you had type 2 diabetes, how much would you experience symptoms from the illness?”). Two of the items assess emotional representations: concern (“If you had type 2 diabetes, how concerned would you be about the illness?”), and emotional response (“If you had type 2 diabetes, how much would the illness affect you emotionally? (e.g. would it make you angry, scared, upset or depressed?)”). One item assesses understanding (“How well do you feel you understand type 2 diabetes?”). Participants were asked to respond to each question on an 11-point response scale, ranging from 0 to 10, with higher scores representing a stronger endorsement of that particular representation. The Brief IPQ has been shown to have good test-retest reliability and to be highly correlated with relevant subscales of the IPQ-R.

| **Table A.** Results of pre-specified exploratory analyses: potential moderators of intervention effects on physical activity | | | |
| --- | --- | --- | --- |
| Measure | Interaction term from analysis of covariance | | |
|  | *df* | *F* | P-value |
| Age | 2, 542 | 0.32 | 0.73 |
| Sex (male/female) | 2, 542 | 3.47 | 0.03 |
| Body mass index | 2, 542 | 0.05 | 0.95 |
| Self-reported Diet | 2, 540 | 0.58 | 0.56 |
| Self-reported weight | 2, 533 | 1.01 | 0.37 |
| Self-rated health | 2, 538 | 0.45 | 0.64 |
| Worry | 2, 540 | 1.99 | 0.14 |
| Anxiety | 2, 507 | 2.00 | 0.14 |
| Behavioral intention |  |  |  |
| Physical activity | 2, 540 | 2.61 | 0.08 |
| Diet | 2, 540 | 0.38 | 0.69 |
| Perceived risk | 2, 535 | 0.42 | 0.65 |
| Response efficacy |  |  |  |
| Physical activity | 2, 539 | 0.79 | 0.45 |
| Diet | 2, 540 | 0.12 | 0.88 |
| Self-efficacy |  |  |  |
| Physical activity | 2, 540 | 1.41 | 0.25 |
| Diet | 2, 540 | 0.07 | 0.94 |
| Perceived severity | 2, 538 | 0.02 | 0.98 |
| Diabetes risk representations |  |  |  |
| Consequences | 2, 542 | 0.32 | 0.73 |
| Timeline | 2, 539 | 0.87 | 0.42 |
| Personal control | 2, 542 | 0.03 | 0.97 |
| Treatment control | 2, 539 | 2.49 | 0.08 |
| Identity | 2, 538 | 0.92 | 0.40 |
| Concern | 2, 540 | 0.21 | 0.81 |
| Understanding | 2, 540 | 1.54 | 0.21 |
| Emotional response | 2, 539 | 0.43 | 0.65 |
| Baseline physical activity | 2, 543 | 2.57 | 0.08 |
| Time since Fenland Study | 2, 542 | 0.30 | 0.74 |
| Risk estimate | 1, 360 | 0.001 | 0.99 |
| All measures were treated continuously unless otherwise specified. | | | |
